# The chromatin remodeller RSF1 is essential for PLK1 deposition and function at mitotic kinetochores

**DOI:** 10.1038/ncomms8904

**Published:** 2015-08-10

**Authors:** Ho-Soo Lee, Yong-Yea Park, Mi-Young Cho, Sunyoung Chae, Young-Suk Yoo, Myung-Hee Kwon, Chang-Woo Lee, Hyeseong Cho

**Affiliations:** 1 Department of Biochemistry, Ajou University School of Medicine, Suwon 443-380, Korea; 2 Department of Biomedical Sciences, Graduate School of Ajou University, Suwon 443-380, Korea; 3 Department of Microbiology, Ajou University School of Medicine, Suwon 443-380, Korea; 4 Department of Molecular Cell Biology, Sungkyunkwan University School of Medicine, Suwon 440-746, Korea

## Abstract

Accumulation of PLK1 at kinetochores is essential for chromosome alignment and segregation; however, the mechanism underlying PLK1 recruitment to kinetochores remains unresolved. The chromatin remodeller RSF1 tightly associates with centromere proteins, but its mitotic function is unknown. Here we show that RSF1 localizes at mitotic kinetochores and directly binds PLK1. RSF1 depletion disrupts localization of PLK1 at kinetochores; the C-terminal fragment of RSF1, which can bind PLK1, is sufficient to restore PLK1 localization. Moreover, CDK1 phosphorylates RSF1 at Ser1375, and this phosphorylation is necessary for PLK1 recruitment. Subsequently, PLK1 phosphorylates RSF1 at Ser1359, stabilizing PLK1 deposition. Importantly, RSF1 depletion mimicks the chromosome misalignment phenotype resulting from PLK1 knockdown; these defects are rescued by RSF1 S1375D or RSF1 S1359D but not RSF1 S1375A, showing a functional link between phosphorylation of RSF1 and chromosome alignment. Together, these data show that RSF1 is an essential centromeric component that recruits PLK1 to kinetochores and plays a crucial role in faithful cell division.

## Abstract (web summary)

Polo-like kinase 1 (PLK1) is recruited to kinetochores during mitosis, where it is required for proper chromosome alignment. Lee *et al*. show that the chromatin-remodelling factor RSF1 is required for PLK1 recruitment, and that this function depends on phosphorylation of RSF1 by the mitotic kinase CDK1.

## Graphical abstract

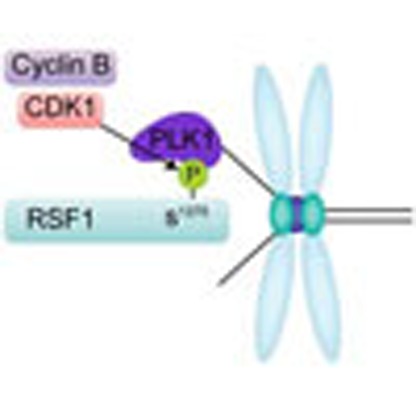

Polo-like kinase 1 (PLK1) is an essential mitotic kinase that controls centrosome maturation and maintenance, microtubule attachment to kinetochores and cytokinesis^1^. Execution of these functions is preceded by dynamic changes in the subcellular localization, abundance and activity of PLK1 at different stages of the cell cycle^2^,^3^. In G_2_ phase, PLK1 first appears at centromeres; later, in mitosis, it becomes enriched at kinetochores. PLK1 at kinetochores stabilizes initial kinetochore–microtubule attachments; consequently, loss of PLK1 function at this stage leads to failures in chromosome alignment^4^,^5^,^6^. Stable microtubule attachments to kinetochores is facilitated by the microtubule-associated proteins CLASP2 and CLIP170 (refs 7, 8), whose phosphorylation and recruitment to the kinetochores are regulated by PLK1. PLK1 also interacts with the key mitotic kinases Aurora B, BubR1 and haspin, and often functions as an upstream kinase^9^,^10^,^11^,^12^,^13^. PLK1 phosphorylates BubR1, and this phosphorylation is important for spindle checkpoint signalling as well as for stable microtubule–kinetochore attachment^9^,^10^. In addition, PLK1-dependent phosphorylation of survivin and haspin contributes to the recruitment of Aurora B to the centromeres^11^,^13^,^14^. At metaphase, ubiquitylation-mediated removal of PLK1 from kinetochores is required for progression into anaphase^15^. Thus, timely positioning of PLK1 at mitotic kinetochores, as well as cooperation between PLK1 and other interacting kinases and phosphatases, enables faithful chromosome alignment and segregation. PLK1-interacting proteins potentially contribute to the localization of PLK1 to kinetochores^7^,^16^,^17^; however, the exact mechanism by which PLK1 accumulates at mitotic kinetochores remains unresolved.

RSF1 is a binding partner of the SNF2H ATPase; together, these proteins form RSF (remodelling and spacing factor), which enforces nucleosome assembly and repositioning^18^,^19^,^20^. Unlike other chromatin-remodelling complexes, RSF1 is found as a component of interphase centromere proteins (CENPs)^21^; in fact, at G_1_ phase, RSF enables assembly of centromeric core nucleosomes containing CENP-A^22^. In addition, RSF1 participates in DNA repair processes by facilitating the assembly of the centromere proteins CENP-S and CENP-X at DNA damage sites^23^,^24^. RSF1 depletion leads to aberrant mitotic progression and chromosome misalignment^22^, suggesting that it plays a regulatory role in mitosis. But to date, this protein's subcellular localization and centromeric function in mitosis remain unknown.

Here we demonstrate that RSF1 localizes at mitotic kinetochores and directly binds PLK1. CDK1-mediated phosphorylation at the C-terminal region of RSF1 provides a docking site for PLK1 and subsequent phosphorylation by PLK1 further stabilizes their interactions. Importantly, RSF1 depletion induces the chromosome misalignment phenotype and these defects are rescued by the phosphomimetic RSF1 mutants. Therefore, RSF1 is a centromeric component that recruits PLK1 to kinetochores in a phosphorylation-dependent manner and is crucial for faithful chromosome alignment.

## Results

### RSF1 directly interacts with PLK1 at mitotic kinetochores

**Figure 1:**
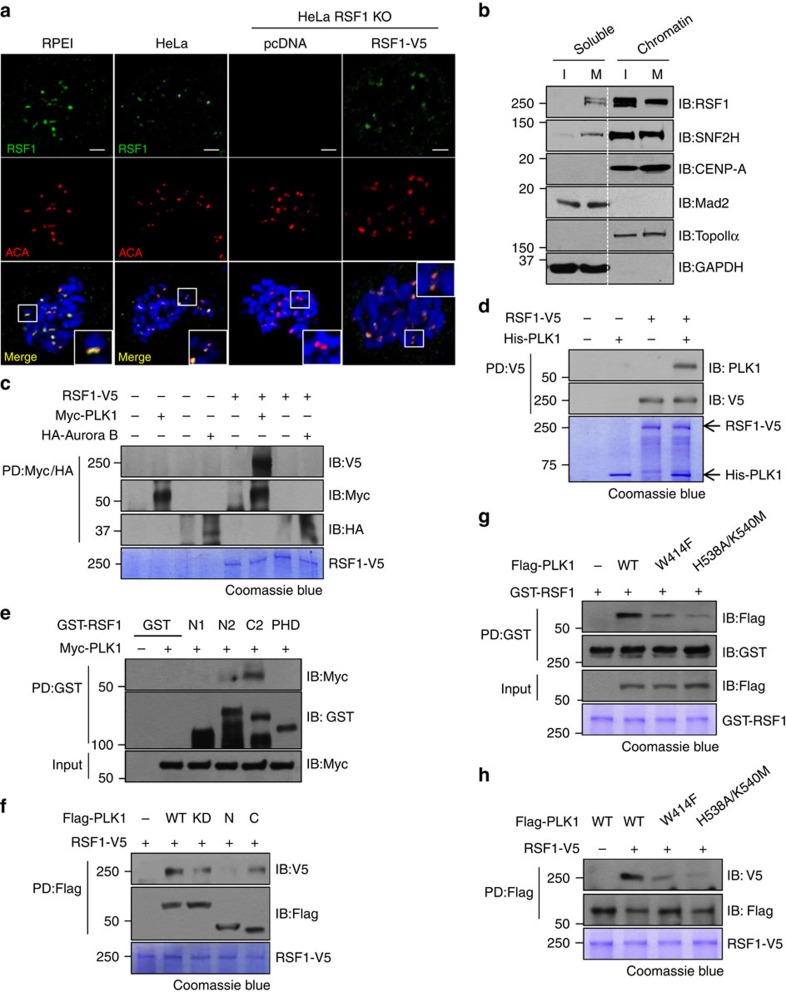
RSF1 localizes at mitotic kinetochores and directly interacts with PLK1. (**a**) RSF1 knockout (KO) HeLa cells were transfected with pcDNA or RSF1-V5. Floating mitotic cells were obtained after nocodazole treatment for 4 h and subjected to chromosome spread immunostaining. Immunofluorescence images of human epithelial RPE1 and HeLa cells are shown: RSF1 (green), ACA (red) and 4,6-diamidino-2-phenylindole (blue). Scale bar, 5 μm. (**b**) Chromatin fractions of asynchronously growing HeLa (I, interphase) and paclitaxel-arrested HeLa cells (M, mitotic phase) were obtained after centrifugation and washing with 0.5 M NaCl. Proteins were eluted as soluble fractions and chromatin-bound fractions were analysed by immunoblotting with the indicated antibodies. Topo IIα and GAPDH were used as controls for chromatin and soluble fractions, respectively. (**c**) *In vitro* binding assays: recombinant RSF1-V5 was incubated with immunopurified Myc-PLK1 or HA-Aurora B for 2 h at 4 °C. RSF1-V5 bound to immobilized Myc-PLK1 or HA-Aurora B was detected by immunoblotting. Recombinant RSF1-V5 was stained with Coomassie blue. (**d**) Recombinant RSF1-V5 was incubated with recombinant His-PLK1 purified from insect cells, and His-PLK1 bound to immobilized RSF1-V5 was detected by immunoblotting. Recombinant RSF1-V5 and His-PLK1 were visualized by Coomassie blue staining. (**e**) Recombinant GST-RSF1 proteins were incubated with Myc-PLK1 expressing mitotic lysates and subjected to immunoblotting. N1: amino acids 1–627, N2: 1–871, C2: 982–1441 and PHD (plant homeodomain): 628–973. (**f**) Immunopurified Flag-PLK1 was incubated with recombinant RSF1-V5. WT, wild type; KD, kinase dead; N, amino acids 1–401; C, amino acids 350–603. (**g**) Recombinant GST-RSF1 proteins were incubated with Flag-tagged PLK1 WT, PLK1-PBD^W414F^ or PLK1-PBD^H538A/K540M^ expressing mitotic lysates and subjected to immunoblotting. Recombinant GST-RSF1 was stained with Coomassie blue. (**h**) *In vitro* binding assays: recombinant RSF1-V5 was incubated with immunopurified Flag-PLK1 WT or Flag-PLK1 PBD mutants for 2 h at 4 °C. RSF1-V5 bound to immobilized Flag-PLK1 WT or Flag-PLK1 PBD mutants was detected by immunoblotting. Recombinant RSF1-V5 was stained with Coomassie blue. See full blots Supplementary Fig. 8.

To investigate the function of RSF1 in mitosis, we first attempted to determine its localization. RSF1 co-stained extensively with anti-centromere antibodies (ACA), a marker of inner kinetochores, on mitotic chromosomes of HeLa cells (Supplementary Fig. 1a); this observation was verified by immunostaining of chromosome spreads of prometaphase-arrested cells. RSF1 co-stained with ACA in HeLa cells, as well as in human epithelial RPE1 cells (Fig. 1a); as expected, the signal disappeared in RSF1 knockout (KO) HeLa cells. Re-expression of RSF1 tagged with V5 (RSF1-V5) in RSF1 KO cells restored RSF1 immunostaining. These data are the first to demonstrate that endogenous RSF1 is localized to mitotic kinetochores.

This result was verified by chromatin fractionation experiments: under our experimental conditions, chromatin-bound proteins remained in the chromatin pellet after a wash with buffer containing 0.5 M NaCl, whereas unstably bound proteins were eluted into soluble chromatin extracts. Accordingly, the outer kinetochore-associated Mad2 was eluted to the soluble fraction, whereas Topo IIα remained in the chromatin pellet (Fig. 1b). The majority of RSF1 and SNF2H remained in the chromatin-bound fraction along with CENP-A, a centromeric nucleosome component, in both interphase and mitotic cells (Fig. 1b). A previous phosphoproteome analysis identified RSF1 as a candidate phosphorylation target of PLK1 (ref. 25), and we observed that RSF1 depletion induced defects in chromosome alignment that were also observed in PLK1-depleted cells (Supplementary Fig. 1b,c)^4^,^5^,^6^. Therefore, we concluded that RSF1 function in mitosis is related to PLK1. Co-immunoprecipitation experiments revealed that endogenous PLK1 co-precipitated with RSF1 in mitotic cells (Supplementary Fig. 2a).

To further test this association, we purified V5-tagged full-length RSF1 protein from HEK293F cells engineered to secrete recombinant RSF1 protein into the culture medium (Supplementary Fig. 2b). When immunopurified Myc-tagged PLK1 and HA-tagged Aurora B obtained from mitotic cells were incubated with recombinant RSF1-V5, RSF1-V5 bound PLK1 but not Aurora B (Fig. 1c). Next, we incubated recombinant RSF1-V5 with recombinant His-PLK1 purified from insect cells; pull-down assays with anti-V5 antibody verified that PLK1 directly bound RSF1 (Fig. 1d), indicating that RSF1 is a bona fide PLK1-interacting protein. It is of note that SNF2H is dispensable for the RSF1–PLK1 interaction. Co-imunoprecipitation assay using RSF1 KO HeLa cell extracts revealed that SNF2H did not co-precipitate with PLK1 (Supplementary Fig. 2c). On pull-down experiments, we verified no interactions between PLK1 and SNF2H (Supplementary Fig. 2d). Next, we generated deletion mutants (Supplementary Fig. 2e) and performed interaction studies with immobilized glutathione *S*-transferase (GST)-tagged RSF1 or FLAG-tagged PLK1. Pull-down assays identified an interaction between the C terminus of RSF1 (RSF1-C2, amino acids 982–1441) and the C terminus of PLK1 (PLK1-C, amino acids 350–603), which contains two Polo-box domains (PBDs) (Fig. 1e,f). A kinase-dead mutant of PLK1 (PLK1^K82R^) retained the ability to bind RSF1; however, both the PLK1-C and PLK1 kinase-dead mutant exhibited reduced binding to RSF1. Because the PBD of PLK1 recognized the phosphopeptide on substrates and Trp414, His538 and Lys540 residues on the PBD are important in the phosphate recognition^26^,^27^, we generated the PLK1-PBD^W414F^ and PLK1-PBD^H538A/K540M^ that were known to disrupt the phosphopeptide-binding activity of the PBD^26^,^28^. Interaction studies with immobilized GST-tagged RSF1 or FLAG-tagged PLK1 revealed that both PLK1-PBD^W414F^ and PLK1-PBD^H538A/K540M^ exhibited a significant loss in their binding to RSF1 (Fig. 1g,h). Thus, the data here clearly showed that RSF1 directly interacts with PLK1 in mitosis.

### RSF1 is crucial for PLK1 deposition at mitotic kinetochores

**Figure 2:**
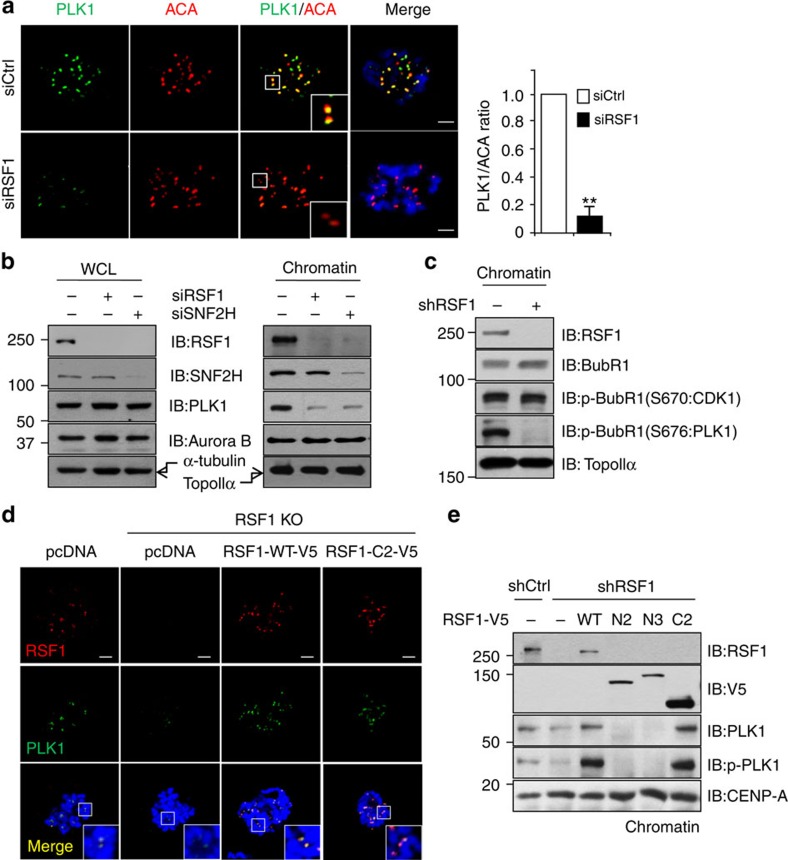
RSF1 depletion impairs kinetochore localization of PLK1 in early mitosis. (**a**) HeLa cells were transfected with siRNAs, and floating mitotic cells were obtained after nocodazole treatment for 4 h and subjected to chromosome spread immunostaining. Images were obtained from representative mitotic cells: PLK1 (green), ACA (red) and 4,6-diamidino-2-phenylindole (DAPI; blue). The graph represents relative intensity of PLK1 against ACA at kinetochores. At least 100 kinetochores of prometaphase cells were analysed in three independent experiments. Data are represented as means±s.e.m. (*n*=3). **P<0.01 versus control siRNA by Student's *t*-test. Scale bar, 5 μm. (**b**) HeLa cells transfected with RSF1 or SNF2H siRNA were treated with paclitaxel for 16 h, and floating mitotic cells were obtained. Whole-cell lysates (WCLs) and chromatin-bound fractions were subjected to immunoblotting. (**c**) Cells stably expressing the RSF1 shRNA plasmid were treated with paclitaxel for 16 h. Chromatin-bound fractions were subjected to immunoblotting. CDK1-specific phosphorylation of BubR1 on Ser670- and PLK1-specific phosphorylation on Ser676 were determined. (**d**) Restoration of PLK1 localization by RSF1: RSF1 KO HeLa cells were transfected with WT-V5 or RSF1-C2-V5 for 48 h. Mitotic cells were analysed by immunofluorescence staining: PLK1 (green), RSF1 (red) or DAPI (blue). Scale bar, 5 μm. (**e**) Immunoblotting of chromatin fractions in RSF1 shRNA cells after reintroduction of RSF1-WT or truncated forms of the *RSF1* gene. See full blots Supplementary Fig. 8.

Because RSF1 binds PLK1 directly, we asked whether RSF1 binding to PLK1 affects PLK1 localization. As shown in Fig. 2a, the kinetochore localization of PLK1 in HeLa cells was significantly diminished in cells treated with RSF1 short interfering RNA (siRNA.) A similar observation was made in immortalized human epithelial RPE1 cells (Supplementary Fig. 3a). In RPE1 cells, complete KO of *RSF1* resulted in inviability (data not shown), whereas RSF1 KO HeLa cells were viable but labile. It is shown that *Rsf1*^*tm1b*^ KO mice in European Mouse Mutant Archive also exhibited complete preweaning lethality. We assumed that one of the main reasons that RSF1 KO HeLa cells are viable is due to low p53 levels, because HeLa cells contain E6 protein targeting p53. In clonogenic survival assay, RSF1 depletion reduced the cell viability ∼30% in HCT116-p53^−/−^cells, whereas ∼80% of HCT116-p53^+/+^ cells died after RSF1 depletion. These data support our hypothesis that functional loss of p53 in RSF1 KO HeLa cells make them viable (Supplementary Fig. 3b,c). In chromatin fractionation experiments, the PLK1 level was also reduced following RSF1 depletion, whereas Aurora B levels remained constant (Fig. 2b, right panel). The reduction of chromatin-bound PLK1 in RSF1-depleted cells was not due to lower overall PLK1 protein levels, because the total protein level of PLK1 in whole-cell lysates remained unchanged (Fig. 2b, left panel). The RSF1 protein level is also reduced in the absence of SNF2H (ref. 19), indicating that SNF2H depletion mimics RSF1 depletion. Next, we investigated whether a reduction in PLK1 levels would affect PLK1-dependent phosphorylation in these cells. BubR1 is phosphorylated by PLK1 at multiple sites, and this phosphorylation is important for the stability of kinetochore–microtubule interactions^9^,^29^. RSF1 depletion did not influence the levels of chromatin-bound BubR1 (Fig. 2c), and CDK1-dependent phosphorylation of BubR1 at Ser670 remained intact. By contrast, the PLK1-specific phosphorylation of BubR1 at Ser676 did not occur in RSF1-depleted cells. These findings were confirmed by immunostaining of chromosome spreads using phospho-specific antibodies against BubR1 (Supplementary Fig. 3d,e). Thus, these data suggest that PLK1-dependent kinetochore function may be also defective in the absence of RSF1.

Given that loss of RSF1 caused reduced PLK1 accumulation at mitotic chromosomes, we predicted that ectopic overexpression of RSF1 in these cells would rescue PLK1 levels. Indeed, expression of RSF1-V5 in RSF1 short hairpin RNA (shRNA) cells increased the amount of chromatin-bound PLK1 (Supplementary Fig. 3f). Likewise, the kinetochore localization of RSF1 and PLK1 in RSF1 KO cells was rescued by overexpression of RSF1-V5 (Fig. 2d). In addition, expression of RSF1-C2^982–1441^, which retains PLK1 binding (Fig. 1e), resulted in accumulation of PLK1 at kinetochores (Fig. 2d) and elevated levels of chromatin-bound PLK1 (Fig. 2e). By contrast, RSF1-N2 and RSF1-N3 mutants neither restored PLK1 localization to kinetochores (Supplementary Fig. 4) nor increased the level of chromatin-bound PLK1 (Fig. 2e). Moreover, phosphorylation of PLK1 at Thr210 in the activation loop^30^ was decreased by RSF1 depletion, and re-expression of RSF1-WT or RSF1-C2 into the RSF1 shRNA cells induced a marked increase in phospho-PLK1 levels. Notably, the RSF1-N2 and RSF1-N3 mutant proteins were still retained in the chromatin-bound fraction. In addition, residual PLK1 levels in RSF1 shRNA cells were almost abolished by re-expression of the RSF1-N2 and RSF1-N3 mutants, suggesting that these deletion mutants may exert dominant-negative effects. Together, these data show that RSF1 binding to PLK1 is necessary for PLK1 deposition on mitotic kinetochores, as well as for its activation.

### CDK1 phosphorylates RSF1

**Figure 3:**
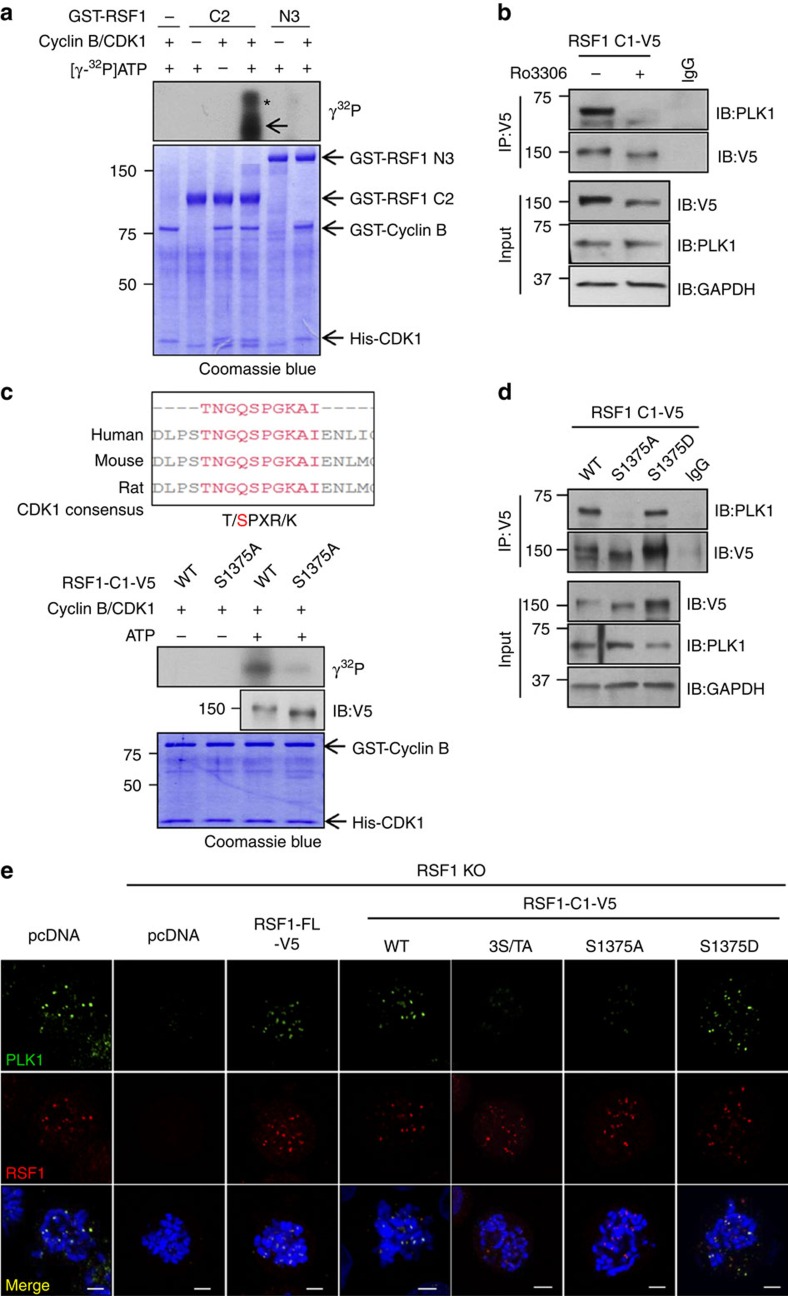
CDK1-mediated phosphorylation at Ser1375 of RSF1 is necessary for PLK1 binding and recruitment to kinetochores. (**a**) *In vitro* kinase assay: recombinant GST-RSF1-C2 or N3 proteins purified in the baculovirus system were incubated with the cyclin B1–CDK1 complex at 30 °C for 30 min in the presence of γ^32^P-ATP. Incorporation of ^32^P into RSF1 protein was visualized by autoradiography. Coomassie blue staining demonstrates equal protein loading. The asterisk indicates the hyper-phosphorylated form of RSF1. (**b**) RSF1 KO cells were transfected with the RSF1-C1-V5 plasmid and treated with paclitaxel for 16 h before harvest. Cells were pre-treated with MG132 for 1 h, and with the CDK1 inhibitor Ro3306 for 15 min. Co-immunoprecipitation experiments were carried out with an anti-V5 antibody. (**c**) Alignment of vertebrate RSF1 with the conserved CDK1 consensus motif (upper panel) and *in vitro* kinase assay of phosphorylation mutants of RSF1-C1. RSF1 KO HeLa cells transfected with the indicated plasmids were treated with paclitaxel for 16 h. RSF1-C1 and RSF1-C1^S1375A^ proteins were immunopurified using anti-V5 antibody and subjected to *in vitro* kinase assays (bottom panel). (**d**) The RSF1-C1^S1375A^ or RSF1-C1^S1375D^ plasmids were transfected into RSF1 KO cells, and mitotic lysates were immunoprecipitated with anti-V5 antibody, followed by immunoblotting. (**e**) RSF1 KO cells were introduced with indicated RSF1 constructs and treated with nocodazole for 4 h. Floating mitotic cells were subjected to chromosome spread immunostaining. Mitotic cells were analysed by immunofluorescence staining: PLK1 (green), RSF1 (red) or 4,6-diamidino-2-phenylindole (blue). Scale bar, 5 μm. See full blots Supplementary Fig. 8.

RSF1-C2, which contains one-third of the C-terminal residues of RSF1, was sufficient to restore PLK1 deposition. Therefore, we next investigated the mechanism by which RSF1 recruits PLK1. The PBD of PLK1 recognizes a phosphopeptide-binding motif in target substrates, whose phosphorylation is often driven by CDK1 (refs 26, 31, 32). Hence we performed *in vitro* kinase assays to investigate whether CDK1 phosphorylates RSF1. Commercially available cyclin B and CDK1 proteins were incubated with purified RSF1 proteins, and phosphorylated proteins were visualized by autoradiography. Under these conditions, both full-length RSF1 (RSF1-FL) and RSF1-C2 were phosphorylated by the CDK1 complex, whereas RSF1-N3 was not phosphorylated (Fig. 3a; Supplementary Fig. 5a). Next, we asked whether CDK1-mediated phosphorylation of RSF1 is important for its interaction with PLK1. To this end, we transfected V5-tagged RSF1-C1^627–1441^ into RSF1 KO cells and treated the transfectants with paclitaxel for 16 h; before harvest, cells were treated for 15 min with the CDK1 inhibitor Ro3306 and the proteasome inhibitor MG132 to prevent mitotic exit. Remarkably, binding of PLK1 to the RSF1 was abolished in cells treated with Ro3306, strongly suggesting that the CDK1-mediated RSF1 phosphorylation is crucial for the PLK1 interaction (Fig. 3b).

In the course of our search for predicted phosphorylation sites in the C terminus of RSF1 using PhosphoMotif Finder (www.hprd.org/PhosphoMotif_finder), we found a large number of mapped phosphorylation sites. Our preliminary mass analysis revealed that three sites in the C terminus of RSF1, Thr1305, Ser1359 and Ser1375, are highly phosphorylated (data not shown). In particular, the CDK1 consensus target site [pT/pSPXR/K] at Ser1375 of RSF1 was well conserved among the human, mouse and rat proteins (Fig. 3c, upper panel). To examine the importance of these sequences, we generated non-phosphorylatable mutants in which a Ser or Thr residue was switched to Ala (T1305A, S1359A and S1375A). In addition, we generated a triple mutant (3S/TA) in which all three Ser and Thr residues were switched to Ala. We first tested whether these mutants differed in their ability to restore PLK1 levels to mitotic chromosomes (Supplementary Fig. 5b). All these point-mutant proteins of RSF1 are well-preserved in the chromatin-bound fractions. Both RSF1-C1^3S/TA^ and RSF1-C1^S1375A^ lost the ability to cause PLK1 to accumulate on mitotic chromosomes, whereas RSF1-C1^T1305A^ and RSF1-C1^S1359A^ still largely retained this ability, suggesting that phosphorylation at Ser1375 is crucial for PLK1 deposition. The importance of the Ser1375 residue was verified by an *in vitro* kinase assay: specifically, RSF1-C2^S1375A^ could not be phosphorylated by the cyclinB1/CDK1 complex (Fig. 3c), indicating that Ser1375 is the major phosphorylation site of CDK1. Next, we examined whether CDK1-mediated phosphorylation at Ser1375 is necessary for PLK1 binding. Co-immunoprecipitation experiments revealed that the RSF1-C2^S1375A^ mutant lost the ability to bind PLK1, whereas RSF1-C1^S1375D^, a phosphomimetic mutant of RSF1 in which Ser1375 was replaced by Asp1375, could bind PLK1 as efficiently as RSF1-C1 (Fig. 3d). Together, these data strongly suggest that phosphorylation of RSF1 at Ser1375 by CDK1 creates a docking site for PLK1.

Next, we performed immunofluorescence staining to examine the ability of these RSF1 phosphorylation mutants to recruit PLK1 to the kinetochores. Concomitant loss of PLK1 and RSF1 in RSF1 KO cells was rescued by re-expression of RSF1-FL or RSF1-C1 (Fig. 3e). Consistent with our findings described above, RSF1-C1^S1375A^ and RSF1-C1^3S/TA^ were not capable of recruiting PLK1, whereas the phosphomimetic RSF1-C1^S1375D^ retained this ability (Fig. 3e). Therefore, we conclude that priming phosphorylation of RSF1 at the site of Ser1375 by CDK1 is necessary for the PLK1 binding, and this RSF1 binding to PLK1 largely contributes to the PLK1 deposition to the kinetochores in early mitosis.

### PLK1 phosphorylates RSF1

**Figure 4:**
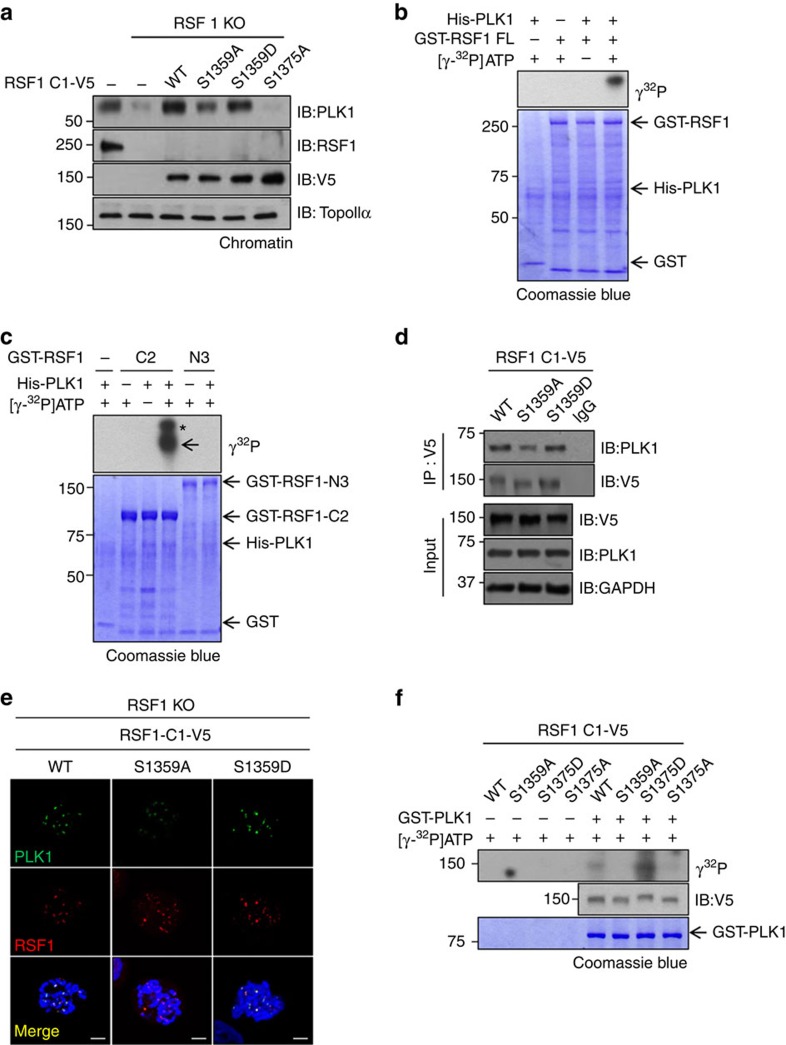
PLK1 phosphorylates the Ser1359 residue of RSF1. (**a**) RSF1 KO HeLa cells were transfected with indicated RSF1-C1 constructs and treated with paclitaxel for 16 h. Chromatin fractions of mitotic cell lysates were analysed by immunoblotting. (**b**,**c**) Recombinant His-PLK1 and GST-RSF1 (FL, C2 and N3) proteins were incubated at 30 °C for 30 min in the presence of γ^32^P-ATP. Incorporation of ^32^P into RSF1 protein was visualized by autoradiography. The asterisk indicates the hyper-phosphorylated form of RSF1-C2. (**d**) The RSF1-C1^S1359A^ or RSF1-C1^S1359D^ plasmids were transfected into RSF1 KO cells, and mitotic lysates were immunoprecipitated with anti-V5 antibody, followed by immunoblotting. (**e**) RSF1 KO cells were introduced with indicated RSF1 constructs and treated with nocodazole for 4 h. Floating mitotic cells were subjected to chromosome spread immunostaining. Scale bar, 5 μm. (**f**) RSF1 KO HeLa cells were transfected with indicated RSF1-C1-V5 constructs and treated with paclitaxel for 16 h. RSF1-C1 protein immunopurified with anti-V5 antibody was incubated with recombinant GST-PLK1 protein in the *in vitro* kinase assay. See full blots Supplementary Fig. 8.

After docking with a substrate, PLK1 can often implement secondary phosphorylation of the substrate^33^,^34^,^35^. In fact, a previous phosphoproteome analysis^25^ identified Ser1359 and Ser1375 of RSF1 as putative phosphorylation sites of PLK1 during mitosis. We noticed that the levels of RSF1-C1^S1359A^ in chromatin fractions were slightly, but consistently, reduced across multiple experiments, whereas those of RSF1-C1^T1305A^ were not (Supplementary Fig. 5b). We reasoned that if the PLK1 levels in the chromatin fraction reflected the stable deposition of PLK1 at the kinetochore, this reduction might reflect a functional role of PLK1 phosphorylation. Consistent with this idea, re-expression of RSF1-C1^S1375A^ in RSF1 KO cells resulted in complete deficiency in PLK1 accumulation, whereas RSF1-C1^S1359A^ partially recovered PLK1 accumulation (Fig. 4a). As expected, both RSF1-C1 and the phosphomimetic RSF1-C1^S1359D^ efficiently increased chromatin-bound PLK1 levels. Next, we determined whether RSF1 could be phosphorylated by PLK1 in *in vitro* kinase assays. Both RSF1-FL and RSF1-C2 were phosphorylated by purified His-PLK1, whereas RSF1-N3 was not (Fig. 4b,c); thus, our data clearly showed that C-terminal RSF1-C2 contains multiple phosphorylation sites for both CDK1 and PLK1. Next, we addressed whether different chromatin deposition of PLK1 in cells expressing RSF1-C1^S1359A^ and RSF1-C1^S1359D^ (Fig. 4a) were due to different binding of these mutants to PLK1. Co-immunoprecipitation experiments revealed that the RSF1-C1^S1359A^ mutant exhibited the reduced binding to PLK1, whereas RSF1-C1^S1359D^ retained its full binding to PLK1 as wild-type PLK1 (Fig. 4d). These findings were also consistent in immuofluorescence staining of chromosome spreads. In RSF1 KO cells, both RSF1-C1^S1359A^ and RSF1-C1^S1359D^ mutants were fully targeted to centromere regions (Fig. 4e). RSF1-C1^S1359A^ conferred inefficient recruitment of PLK1 to kinetochores, whereas wild-type RSF1-C1 and the phosphomimetic RSF1-C1^S1359D^ fully retained the ability of PLK1 recruitment, suggesting that the PLK1-induced phosphorylation at Ser1359 is also important for the PLK1–RSF1 interaction.

In addition, we investigated how CDK1 and PLK1 cooperate with each other in RSF1 phosphorylation. Importantly, the phosphomimetic RSF1-C1^S1375D^ was highly phosphorylated by PLK1, whereas RSF1-C1 was phosphorylated to a lesser extent (Fig. 4f). By contrast, neither RSF1-C1^S1359A^ nor RSF1-C1^S1375A^ was phosphorylated by PLK1. Together, these data suggest that Ser1359 of RSF1 is the phosphorylation site of PLK1. Loss of CDK1-mediated phosphorylation at Ser1375 of RSF1 barely triggered PLK1-mediated secondary phosphorylation. In that sense, the phosphomimetic RSF1-C1^S1375D^ serves as an efficient phosphorylation substrate for PLK1. It is worth mentioning that in an *in vitro* kinase assay reaction containing sufficient amounts of enzyme and substrates, PLK1 might phosphorylate substrates through a self-recruitment mechanism^36^. Taken together, these data indicate that priming phosphorylation of Ser1375 of RSF1 by CDK1 facilitates the subsequent phosphorylation by PLK1 at Ser1359, and that this PLK1-mediated phosphorylation contributes to stable deposition of PLK1 to the mitotic chromosomes.

### Chromosome alignment defects in RSF1 KO cells

Loss of PLK1 activity in early mitosis leads to unaligned chromosomes because kinetochore–microtubule attachments are not stably formed^4^,^5^,^6^. Our group and others^22^ showed that RSF1-deficient cells suffer from chromosome-alignment defects. Therefore, we investigated whether chromosome-alignment defects in RSF1-deficient cells are derived from deficiencies in PLK1 deposition and function. In fact, in a cold-stable microtubule assay, RSF1-depleted cells also did not form stable microtubule–kinetochore attachments (Supplementary Fig. 6).

**Figure 5:**
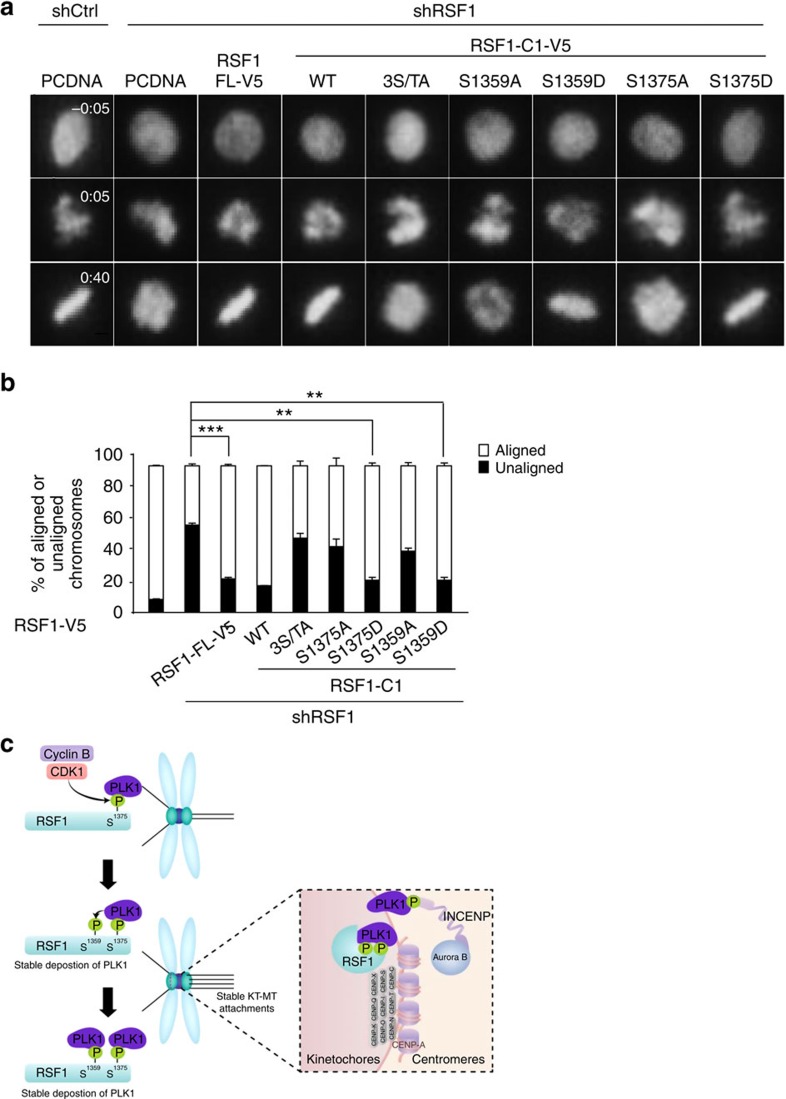
RSF1-deficient cells suffering from chromosome-alignment defects are rescued by RSF1 that retains the ability to bind PLK1. (**a**) After RSF1 shRNA cells stably expressing the RSF1 shRNA plasmid were co-transfected with H2B-GFP and indicated RSF1-V5 constructs, cells were synchronized at the G_1_/S boundary by adding 2 mM of thymidine for 24 h and released from the thymidine block for 8 h. Time-lapse images were acquired every 5 min. Scale bar, 5 μm. (**b**) Quantification of aligned or unaligned chromosome (%) in the acquired images in **a**. At least 30 cells on average were traced in three independent experiments (*n*=30). Data are represented as means±s.e.m. (*n*=3). **P<0.01, ***P<0.001 by Student's *t*-test. (**c**) A proposed model for recruitment of PLK1 to the kinetochore by RSF1.

Next, we monitored mitotic progression by monitoring green fluorescent protein (GFP)-fused histone H2B (GFP-H2B) using time-lapse microscopy. Because RSF1 shRNA cells are sensitive to external stresses, we monitored them under low magnification. More than 90% of control shRNA cells exhibited proper chromosome alignment during mitotic progression, whereas RSF1 shRNA cells consistently exhibited profound defects in chromosome alignment (Fig. 5a). Up to 60% of RSF1 shRNA cells experienced severe chromosome misalignment, and this phenotype was corrected by ectopic overexpression of RSF1-FL or RSF1-C1. Importantly, all the phospho-dead mutants (RSF1-C1^3S/TA^, RSF1-C1^S1375A^ and RSF1-C1^S1359A^) failed to correct the chromosome-alignment defects (Fig. 5a,b), revealing an intimate functional link between chromosome alignment and PLK1 deposition in RSF1-depleted cells. Likewise, both RSF1-C1^S1375D^ and RSF1-C1^S1359D^ were as efficient as RSF1-C1 in correcting chromosome-alignment defects: in all cases, the frequency of unaligned chromosomes was reduced to 15–20% (Fig. 5b). Although RSF1-C1^S1359A^ could cause partial accumulation of PLK1 on mitotic chromosomes (Fig. 4a), it failed to correct chromosome-alignment defects (Fig. 5a,b). Thus, these data suggest that correction of alignment defects in RSF1-depleted cells requires full restoration of PLK1 levels at mitotic kinetochores. Together, these data indicate that the chromosome-alignment defects resulting from RSF1 depletion are due to loss of PLK1 function at mitotic kinetochores. Phosphorylation of RSF1 by CDK1 and PLK1 confers stable binding on PLK1, which is necessary for PLK1 function at mitotic kinetochores.

## Discussion

In this study, we discovered a novel function of RSF1 in mitosis. RSF1 is a component of a chromatin remodeller, whose primary function is facilitating nucleosome assembly and repositioning. In addition to its function as a chromatin remodeller, RSF1 plays a crucial role in PLK1 recruitment and deposition on mitotic kinetochores. On the basis of our findings, we propose a model (Fig. 5c) in which RSF1 locates at the kinetochore proximal to centromeres in early mitosis. Active CDK1 phosphorylates Ser1375 of the RSF1, which provides the docking site for PLK1. The PBD of PLK1 binds to the phosphorylated Ser1375 of RSF1, and this interaction facilitates a subsequent PLK1-mediated phosphorylation on the Ser1359 residue, which further promotes and stabilizes PLK1 deposition. Full PLK1 deposition at the kinetochores promotes the formation of stable kinetochore–microtubule attachments through phosphorylation of multiple target substrates. Another interacting partner of PLK1, the inner centromeric protein INCENP, is included in this model. It is of worthwhile mentioning that kinetochore localization of RSF1 in early mitosis is not related to centromere proteins, CENP-S/X^23^,^24^. Immunofluorescence staining showed that depletion of CENP-S/X did not affect the localization of RSF1 and PLK1 at centromeres (Supplementary Fig. 7a–d).

PLK1 plays multiple functions in mitotic progression; thus, it is important to understand the mechanism by which PLK1 is recruited to the kinetochores. In G_2_ phase, PBIP1 (also known as CENP-U) recruits PLK1 to centromeres, and the docking site for PLK1 is created by self-priming phosphorylation of PLK1 on PBIP1 (ref. 36). In early mitosis, PLK1 induces PBIP1 degradation; however, PLK1 is retained at the kinetochores even after degradation of PBIP1. Thus, the PLK1 population freed from the PBIP1–PLK1 complex must interact with other interacting partners to maintain its position at the kinetochores^36^,^37^,^38^. Other kinetochore/centromere-associated proteins, such as Bub1 kinase^28^ and INCENP^39^, contribute to PLK1 localization at kinetochores. INCENP, a component of the chromosomal passenger complex (CPC), is likely to be a scaffold protein for CPC components^40^,^41^, and the C terminus of INCENP also provides a binding site for Aurora B^42^,^43^. Recruitment of the CPC to centromeres involves a series of structural and functional interactions among these proteins and other mitotic kinases such as PLK1, Bub1 kinase^28^ and others^11^,^39^. PLK1-dependent phosphorylation of survivin and haspin also contributes to the recruitment of Aurora B to the centromeres^11^,^14^. In addition, other PLK1-interacting proteins that are associated with microtubules also affect PLK1 recruitment to the kinetochores^7^,^8^. Thus, these PLK1-interacting proteins mutually affect one another's localization and function; consequently, it remains unclear whether the initial recruitment of PLK1 is achieved by these proteins. In addition, it remains unknown whether these proteins affect one another through direct interactions. Accordingly, it is difficult to exclude the possibility that deletion of these proteins affects PLK1 localization indirectly, either through other mediator proteins or by altering kinetochore/centromere structure. Here we propose that RSF1 is the crucial PLK1 regulatory molecule at kinetochores, and that it differs from other PLK1-interacting proteins. RSF1 is tightly associated with centromere proteins, and its localization is not affected by PLK1 binding. Notably, the RSF1-N2 and RSF1-N3 mutants, and the non-phosphorylatable mutants RSF1^3S/TA^ and RSF1^S1375A^, all of which lack the ability to bind PLK1, were still retained in the chromatin-bound fraction (Fig. 2e) and at mitotic kinetochores (Fig. 3e); therefore, RSF1 deposition on mitotic kinetochores does not rely on the PLK1 binding. In addition, depletion of RSF1 did not disturb other kinetochore/centromere-associated proteins. Depletion of RSF1 changed neither the chromatin-bound levels of BubR1 (Fig. 2c) nor the overall level of Bub1 (data not shown). Likewise, RSF1 depletion did not disturb the CPC at the centromeres: chromatin-bound levels of INCENP, survivin and Aurora B remained unchanged in RSF1-depleted cells (Fig. 2b; Supplementary Fig. 7e,f). Finally, RSF1 not only co-localizes with PLK1 but is also a direct binding partner of PLK1 (Fig. 1). All these findings lead us to conclude that RSF1 is the ‘missing piece' of the PLK1 regulatory machinery.

In this study, we utilized chromatin fractionation experiments to quantitatively trace the changes in kinetochore/centromere protein levels. We noticed that a portion of residual PLK1 in the chromatin-bound fraction was still observed in RSF1 KO HeLa cells (Fig. 4a), suggesting that other PLK1-binding partners also contribute to PLK1 deposition. Because RSF1 localizes near the centromeres, we examined whether RSF1 cooperated with INCENP in PLK1 deposition. Depletion of INCENP by RNA interference did not alter the chromatin-bound RSF1 level, and siRNAs against either RSF1 or INCENP reduced the level of chromatin-bound PLK1. Notably, double knockdown of RSF1 and INCENP caused a further reduction in PLK1 levels (Supplementary Fig. 7g), suggesting that RSF1 cooperates with INCENP to stably position PLK1. Because Bub1 kinase^28^ was also shown to affect PLK1 localization, we examined whether the reduction in PLK1 in RSF1 KO cells could be mediated by the changes in Bub1 level. We found that RSF1 depletion did not affect the Bub1 localization at kinetochores (Supplementary Fig. 7h,i). In addition, it is worth noting that residual PLK1 levels in cells expressing RSF1 shRNA cells were almost abolished by re-expression of the RSF1-N2 and RSF1-N3 mutants (Supplementary Fig. 4). Because these mutants remained in the chromatin-bound fraction (Fig. 2e), we interpreted these data to mean that the deletion mutants exerted a dominant-negative effect on endogenous RSF1. Likewise, the residual PLK1 level in RSF1 KO cells was further reduced after re-expression of RSF1^S1375A^ (Fig. 4a).

Localization of PLK1 is often mediated through interactions of the PBD with proteins that contain its consensus binding phosphopeptide [S-pT/pS-P/X] and are primed by CDK1 or self-primed by PLK1 (refs 26, 30, 31, 32). Thus, PLK1-docking sites largely overlap with consensus sites for CDK1 phosphorylation except that the PBD-binding sites usually contain the Ser at the −1 position. The data here also suggest that RSF1 contains at least two phosphorylation sites; the first phosphorylation site of Ser1375 resides with the [Q-pS-P-X-K] sequences that perfectly match with the CDK1 substrate consensus [pT/pS-P-X-R/K], and, thus, this site provides an optimal site for priming phosphorylation by CDK1 and creates for the PLK1 docking, although it lacks the Ser at the −1 position. The second phosphorylation site of Ser1359 contains [G-pS-P-X-X] that is less conserved for the CDK1 substrate and phosphorylated by PLK1. The RSF1–PLK1 interaction occurred in a phosphorylation-dependent manner because PLK1-PBD^W414F^ and PLK1-PBD^H538A/K540M^ mutants that lose the phosphopeptide-binding activity exhibited a significant reduction in the binding to RSF1 (Fig. 1g). Likewise, the phosphorylation-defective RSF1^S1375A^ mutants completely lost the binding to PLK1 (Figs 3d and 4a), whereas RSF1^S1359A^ also exhibited a reduced binding to PLK1 to a lesser extent (Fig. 4e). In addition, these phosphorylation processes must occur in order because the RSF1^S1375A^ mutant (which has lost the priming phosphorylation) is no longer phosphorylated by PLK1 (Fig. 4d). Thus, PLK1 docking to the phosphorylated Ser1375 instigates PLK1-induced phosphorylation at Ser1359. Furthermore, the recruitment and maintenance of PLK1 via phosphorylation of RSF1 is physiologically important because the non-phosphorylatable mutants of RSF1^S1375A^ and RSF1^S1359A^ failed to align chromosomes in RSF1-depleted cells (Fig. 5a,b). RSF1^S1375A^ almost completely lost its ability to accumulate the PLK1, whereas RSF1-C1^S1359A^ was partially capable of causing PLK1 accumulation on mitotic chromosomes (Fig. 4a). Thus, it is likely that correction of the alignment defects in RSF1-depleted cells requires full deposition of PLK1 through phosphorylation by both CDK1 and PLK1. In these cells, the PLK1-mediated phosphorylation on BubR1, which is important for stable kinetochore–microtubule interactions^9^,^29^, was also defective (Fig. 3b,c), aggravating chromosome misalignment. Thus, we here propose that RSF1 is essential for proper kinetochore function in early mitosis. Notably, a great deal of recent work has focused on the upstream regulatory function of PLK1 on mitotic kinases and proteins such as the Mis18 complex and CENP-A at centromeres^14^,^44^,^45^. In particular, RSF1 is involved in CENP-A deposition at early G1 phase, and future studies should investigate whether RSF1 cooperates with PLK1 in CENP-A deposition.

## Methods

### Cell culture and treatments

HeLa (American Type Culture Collection (ATCC), CCL-2) and HEK293T (ATCC, ACS-4500) cell lines were maintained in low-glucose and high-glucose DMEM supplemented with 10% fetal bovine serum (Invitrogen), respectively. The hTERT-immortalized retinal pigment epithelial cell line, RPE1, was purchased from ATCC (CRL-4000) and maintained in F12 medium with fetal bovine serum in the presence of hygromycin B. The HeLa cells stably expressing RSF1 shRNA targeting 3′-untranslated region of *RSF1* gene was established by infection of the lentiviral vector containing RSF1 shRNA, followed by clonal selection in the presence of puromycin (1 μg ml^−1^) according to the manufacture's protocol (Sigma-Aldrich). To obtain cells synchronized at prometaphase, cells were treated with 100 ng ml^−1^ of paclitaxel (Sigma-Aldrich) or 100 nM of nocodazole (Sigma-Aldrich) for 12–16 h and collected by gentle shake-off.

### Generation of RSF1 KO cells

To generate RSF1 KO cells, TALEN (transcription activator-like effector nuclease) plasmids^46^ targeting the *RSF1* gene were engineered by ToolGen Inc. (Korea). TALENs induce site-specific double-strand breaks, which leads to frameshift mutation at the *RSF1* gene. Among them, the most effective RSF1-specific TALEN plasmid targeted the exon 3 of *RSF1* gene and contained the sequences of 5′-TGCGTCTCCAGCCAATTGGT-3′ (TALEN-L) and 5′-TGGTACCAGTACATGAGGCC-3′ (TALEN-R) that are linked by the endonuclease (EN) target sequence. To enrich the RSF1 KO cells, the pRG2S surrogate reporter plasmids^47^ were co-transfected into HeLa cells using Lipofectamine 2000. The pRG2S reporters are composed of genes encoding two fluorescent proteins (red and green) and the frameshift mutations by EN is designed to restore the GFP gene. At 48 h post-transfection, the transfected cells expressing green fluorescence were sorted using fluorescence-activated cell sorting (FACS Vantage, BD Biosciences) and seeded onto plates according to the manufacturer's protocol. Two weeks later, the cells were collected to determine the expression levels of RSF1 protein by immunoblot. RSF1 KO candidate cells were reseeded on the 96-well plates with serial dilution to obtain a single-cell-derived colony.

### Transfection and RNA interference

The transfection of plasmids or siRNA oligonucleotides was carried out using polyethylenimine (Polysciences) or Lipofectamine 2000 (Invitrogen) as previous described^48^. The siRNA oligonucleotide sequences are as follows: human RSF1 #1, 5′-UCGAAACGAGUUGGCUGAGACUCUU-3′; RSF1 #2, 5′-GGAAAAUGUCAAACCCAUU-3′; and human SNF2H, 5′-ATAGCTCTTCATCCTCCTCTT-3′. The lentiviral vector expressing RSF1 shRNA that targets 3′-UTR of *RSF1* gene contains the sequences of 5′-CCGGGTGCTAATTTATTCCACGGTACTCGAGTACCGTGGAATAAATTAGCACTTTTTG-3′.

### Plasmids and purification of recombinant proteins

Plasmids encoding V5-tagged full length and deletion mutants of RSF1 (N2, N3, C1 and C2) were kindly provided by Dr Ie-Ming Shin (Johns Hopkins Medical School). Flag-tagged full length and deletion constructs of PLK1 as well as recombinant baculoviruses of PLK1 (His-PLK1 and GST-PLK1) were kindly provided by Dr Young-Joo Jang (Dankook University). Site-directed mutagenesis was carried out on the RSF1-V5 C1 plasmid using Muta-Direct Site Directed Mutagenesis Kit (iNtRON Biotechnology) to change Thr1305, Ser1359 and Ser1375 to Ala or Asp (T1305A, S1359A, S1359D, S1375A and S1375D). Recombinant baculoviruses of GST-RSF1 WT, C2 and N3 were created using Gateway system (Invitrogen). Briefly, RSF1 complementary DNA was subcloned into the pDEST20 destination vector using LR Clonase (Invitrogen). The pDEST20-RSF1 bacmid was transfected into Sf9 cells and the supernatant containing recombinant virus (GST-RSF1) was collected after 4 days. In addition, V5-tagged RSF1 complementary DNA was subcloned into the DraIII/BsiWI sites of the pKVL plasmid that contains an endoplasmic reticulum (ER) leader sequence^49^. To obtain recombinant V5-RSF1 protein in mammalian cells, 2 μg of the pKVL-V5-RSF1 plasmid was transfected to 100 ml of FreeStyle 293-F cells. After 5–7 days, the culture supernatant was harvested by a centrifugation, concentrated using Centricon Plus-70 Centrifugal Filter (Millipore). V5-RSF1 protein was purified using anti-V5 antibody (Invitrogen, R960-25) immobilized to Sepharose, if necessary.

### Immunoprecipitation and *in vitro* binding assays

For immunoprecipitation, cells were lysed using a sonicator (EpiShear Probe Sonicator, Active motif, 120 W, 20 KHz) in E1A buffer (50 mM Tris-HCl (pH 7.4), 150 mM NaCl, 0.1% NP-40, 1 mM dithiothreitol, 5 mM EDTA) containing protease and phosphatase inhibitors (1 mg ml^−1^ aprotinin, 1 mg ml^−1^ leupeptin, 5 mM NaF and 0.5 mM Na_3_VO_4_). Protein lysates were immunoprecipitated with indicated antibodies for 12 h at 4 °C under constant rotation. The protein–antibody complex was further incubated with protein A-Sepharose beads (GE Helathcare) for 1 h 30 min at 4 °C and the immune complex were washed and subjected to immunoblotting. For immunoblotting, the following antibodies were used: mouse anti-RSF1 (Upstate, 05-727); rabbit anti-PLK1 (Santa Cruz Biotechnology, sc5585) and mouse anti-Aurora B (BD science, 611083); mouse anti-Flag (M2 Flag sigma, F3165); mouse-Myc (Santa Cruz Biotechnology, sc-40); and mouse HA (Santa Cruz Biotechnology, sc-7392) antibodies. For *in vitro* binding assays, Flag-PLK1, Myc-PLK1 and HA-Aurora B proteins were immunopurified from mitosis-enriched HEK293T cells. Recombinant proteins of His-PLK1, GST-PLK1, GST-RSF1 and RSF1-V5 were prepared as described in the above. The proteins were incubated for 2 h at 4 °C under constant rotation and beads-bound immune complexes were washed for four times and subjected to immunoblot analysis.

### *In vitro* kinase assay

Recombinant GST-fused RSF1 proteins (RSF1-FL, RSF1-C2 and RSF1-N3) or immunopurified V5-RSF1 proteins (RSF-C1, RSF1-C1^S1359A^, RSF1-C1^S1375A^ and RSF1-C1^S1375D^) were incubated with either human GST-Cyclin B1/GST-Cdk1 (Cell Signaling Technology) or recombinant His/GST-PLK1 proteins in the kinase buffer (20 mM HEPES, 0.14 M NaCl, 3 mM KCl, 5 mM MgCl_2_, pH 7.4) with 2 μCi of γ^32^P-ATP (PerkinElmer) for 30 min at 30 °C. The reactions were terminated by adding 6 × SDS sample buffer followed by heating to 100 °C for 5 min. The proteins were separated on gradient SDS–polyacrylamide gel, and the incorporation of ^32^P was visualized by autoradiography.

### Cell fractionation

Mitotic cells were lysed with fractionation buffer I (10 mM Tris (PH 8.0), 25 mM KCl, 5 mM MgCl2, 0.5% NP-40, 1 mM dithiothreitol, 1 mg ml^−1^ aprotinin, 1 mg ml^−1^ leupeptin, 5 mM NaF and 0.5 mM Na_3_VO_4_) and incubated on ice for 5 min. The supernatant (S1) and pellet (P1) were obtained after centrifugation at 1,300*g* for 5 min at 4 °C. The S1 fraction was refined by high-speed centrifugation at 20,000*g* for 10 min at 4 °C and the supernatant was used as a soluble cytosolic fraction (S2). The insoluble pellet (P1) was washed twice and lysed with fractionation buffer II (10 mM Tris (PH 8.0), 500 mM NaCl, 0.1% NP-40, 5 mM EDTA, 1 mg ml^−1^ aprotinin, 1 mg ml^−1^ leupeptin, 5 mM NaF and 5.5 mM Na_3_VO_4_) by sonication (Active motif, 120 W, 20 KHz). After centrifugation at 1,700*g* for 5 min at 4 °C, the chromatin-bound nuclear fraction (supernatant) was obtained. The concentrations of lysates were normalized by Bradford assay (Bio-Rad Laboratories), and lysates were analysed by immunoblotting. The antibodies used were as follows: mouse anti-BubR1 (BD science, 612505, 1:1,000 in TBST (Tris-buffered saline+Tween 20) with 1% bovine serum albumin, BSA); mouse anti-RSF1 (Upstate, 05-727, 1:1,000 in TBST with 1% BSA); rabbit anti-pBubR1 (gift from Chang-Woo Lee, 1:3,000 in TBST with 1% BSA); mouse anti-INCENP (Abcam, ab36453, 1:1,000 in TBST with 1% BSA); rabbit anti-BubR1 (Cell signaling Technology, #2186, 1:1,000 in TBST with 1% BSA); and rabbit anti-topoisomerase IIα (Santa Cruz Biotechnology, sc-13058, 1:1,000 in TBST with 1% BSA).

### Chromosome spreading

HeLa cells were treated with 100 ng ml^−1^ of nocodazole for 4 h and floating mitotic cells were collected by gentle shake-off. The cells were incubated in KCl (75 mM) buffer for 10 min at room temperature and centrifuged at 1,300*g* for 5 min using Cytospin (Hanil Science, Korea). The cells were fixed with 4% paraformaldehyde for 15 min and then permeabilized with 0.5% Triton X-100 for 10 min. The fixed cells were blocked with 3% BSA for 1 h at room temperature. The antibodies were used mouse anti-RSF1 (Upstate, catalog, 1:100 in PBS with 3% BSA) and human anti-ACA (Immunovision, HCT-0100, 1:2,000 in PBS with 3% BSA).

### Immunocytochemistry

Mitosis-arrested HeLa cells after nocodazole (100 ng ml^−1^) treatment were fixed with 4% paraformaldehyde in PBS at pH 7.4 for 10 min. The fixed cells were permeabilized with 0.5% Triton X-100 for 10 min and blocked with 3% BSA in PBS for 1 h, followed by overnight incubation with appropriate primary antibodies at 4 °C. Primary antibody-incubated cells were washed three times with PBS and incubated with fluorescence-conjugated secondary antibody for 1 h. After washing off the secondary antibody-incubated cells, the nuclei were stained with 4,6-diamidino-2-phenylindole (1:50,000, Molecular Probes) for 10 min, followed by washing five times with 0.1% Triton X-100. Cells were mounted with Vectashield (Vector Laboratories). The following antibodies were used: mouse anti-RSF1 (Upstate, 05-727, 1:500 in PBS with 3% BSA); mouse anti-RSF1 (Abcam, ab109002, 1:500 in PBS with 3% BSA); mouse anti-α-tubulin (Abcam, ab7291, 1:500 in PBS with 3% BSA); mouse anti-PLK1 (Abcam, 17057, 1:500 in PBS with 3% BSA); rabbit anti-PLK1 (Santa Cruz Biotechnology, sc5585, 1:500 in PBS with 3% BSA); and human anti-ACA (Immunovision, HCT-0100, 1:2,000 in PBS with 3% BSA). For image acquisition, Nikon A1R-A1 Confocal Microscope system with 60 × 1.4 numerical aperture (NA) Plan-Apochromat objective (Nikon Instrument Inc.) or LSM710 with 63 × 1.4 NA Plan-Apochromat objective (Carl Zeiss Zeiss) were used and analysed by the NIS elements C program or the ZEN 2011 program, respectively.

### Live-cell imaging

HeLa cells transfected with GFP-H2B and other appropriate plasmids were synchronized at the G_1_/S boundary by the double thymidine block method as described previously^50^. At 8 h after release from the thymidine block, the cells were treated with 9 μM of RO3306 for 2 h to arrest cells at G_2_ phase. The synchronized HeLa cells at G_2_ phase regained the cell cycle progression in a microscope stage incubator at 37 °C in a humidified atmosphere of 5% CO_2_ throughout the experiment. Fluorescence images were acquired every 5 min using a Nikon eclipse Ti with a 20 × 1.4 NA Plan-Apochromat objective. Images were captured with an iXonEM +897 Electron Multiplying charge-coupled device camera and analysed using NIS elements Ar microscope imaging software.

### Statistical analysis

In each result, the error bars represent the mean±s.e.m. from at least three independent experiments. The statistical significance was determined with two-sided unpaired Student's *t*-test. *P* values are indicated in the legends.

## Additional information

**How to cite this article:** Lee, H.-S. *et al*. The chromatin remodeller RSF1 is essential for PLK1 deposition and function at mitotic kinetochores. *Nat. Commun.* 6:7904 doi: 10.1038/ncomms8904 (2015).

## Supplementary Material

Supplementary Information Supplementary Figures 1-8

## References

[b1] Petronczki M., Lenart P. & Peters J. M. Polo on the rise-from mitotic entry to cytokinesis with Plk1. Dev. cell 14, 646–659 (2008). 18477449 10.1016/j.devcel.2008.04.014

[b2] Barr F. A., Sillje H. H. & Nigg E. A. Polo-like kinases and the orchestration of cell division. Nat. Rev. Mol. Cell Biol. 5, 429–440 (2004). 15173822 10.1038/nrm1401

[b3] Zitouni S., Nabais C., Jana S. C., Guerrero A. & Bettencourt-Dias M. Polo-like kinases: structural variations lead to multiple functions. Nat. Rev. Mol. Cell Biol. 15, 433–452 (2014). 24954208 10.1038/nrm3819

[b4] Sumara I. . Roles of polo-like kinase 1 in the assembly of functional mitotic spindles. Curr. Biol. 14, 1712–1722 (2004). 15458642 10.1016/j.cub.2004.09.049

[b5] Lenart P. . The small-molecule inhibitor BI 2536 reveals novel insights into mitotic roles of polo-like kinase 1. Curr. Biol. 17, 304–315 (2007). 17291761 10.1016/j.cub.2006.12.046

[b6] Li H. . Phosphorylation of CLIP-170 by Plk1 and CK2 promotes timely formation of kinetochore-microtubule attachments. EMBO J. 29, 2953–2965 (2010). 20664522 10.1038/emboj.2010.174 PMC2944045

[b7] Maia A. R. . Cdk1 and Plk1 mediate a CLASP2 phospho-switch that stabilizes kinetochore-microtubule attachments. J. Cell Biol. 199, 285–301 (2012). 23045552 10.1083/jcb.201203091 PMC3471233

[b8] Kakeno M. . Plk1 phosphorylates CLIP-170 and regulates its binding to microtubules for chromosome alignment. Cell Struct. Funct. 39, 45–59 (2014). 24451569 10.1247/csf.14001

[b9] Elowe S., Hummer S., Uldschmid A., Li X. & Nigg E. A. Tension-sensitive Plk1 phosphorylation on BubR1 regulates the stability of kinetochore microtubule interactions. Genes Dev. 21, 2205–2219 (2007). 17785528 10.1101/gad.436007 PMC1950859

[b10] Huang H. . Phosphorylation sites in BubR1 that regulate kinetochore attachment, tension, and mitotic exit. J. Cell Biol. 183, 667–680 (2008). 19015317 10.1083/jcb.200805163 PMC2582891

[b11] Chu Y. . Aurora B kinase activation requires survivin priming phosphorylation by PLK1. J. Mol. Cell Biol. 3, 260–267 (2011). 21148584 10.1093/jmcb/mjq037 PMC3150119

[b12] Carmena M., Wheelock M., Funabiki H. & Earnshaw W. C. The chromosomal passenger complex (CPC): from easy rider to the godfather of mitosis. Nat. Rev. Mol. Cell Biol. 13, 789–803 (2012). 23175282 10.1038/nrm3474 PMC3729939

[b13] Ghenoiu C., Wheelock M. S. & Funabiki H. Autoinhibition and Polo-dependent multisite phosphorylation restrict activity of the histone H3 kinase Haspin to mitosis. Mol. Cell 52, 734–745 (2013). 24184212 10.1016/j.molcel.2013.10.002 PMC3865225

[b14] Zhou L., Tian X., Zhu C., Wang F. & Higgins J. M. Polo-like kinase-1 triggers histone phosphorylation by Haspin in mitosis. EMBO reports 15, 273–281 (2014). 24413556 10.1002/embr.201338080 PMC3989693

[b15] Beck J. . Ubiquitylation-dependent localization of PLK1 in mitosis. Nat. Cell Biol. 15, 430–439 (2013). 23455478 10.1038/ncb2695 PMC7116173

[b16] Amin M. A., Itoh G., Iemura K., Ikeda M. & Tanaka K. CLIP-170 recruits PLK1 to kinetochores during early mitosis for chromosome alignment. J. Cell Sci. 127, 2818–2824 (2014). 24777477 10.1242/jcs.150755

[b17] Qi W., Tang Z. & Yu H. Phosphorylation- and polo-box-dependent binding of Plk1 to Bub1 is required for the kinetochore localization of Plk1. Mol. Biol. Cell 17, 3705–3716 (2006). 16760428 10.1091/mbc.E06-03-0240 PMC1525235

[b18] LeRoy G., Orphanides G., Lane W. S. & Reinberg D. Requirement of RSF and FACT for transcription of chromatin templates in vitro. Science 282, 1900–1904 (1998). 9836642 10.1126/science.282.5395.1900

[b19] Loyola A. . Functional analysis of the subunits of the chromatin assembly factor RSF. Mol. Cell. Biol. 23, 6759–6768 (2003). 12972596 10.1128/MCB.23.19.6759-6768.2003 PMC193931

[b20] Loyola A., LeRoy G., Wang Y. H. & Reinberg D. Reconstitution of recombinant chromatin establishes a requirement for histone-tail modifications during chromatin assembly and transcription. Genes Dev. 15, 2837–2851 (2001). 11691835 10.1101/gad.937401 PMC312801

[b21] Obuse C. . Proteomics analysis of the centromere complex from HeLa interphase cells: UV-damaged DNA binding protein 1 (DDB-1) is a component of the CEN-complex, while BMI-1 is transiently co-localized with the centromeric region in interphase. Genes Cells 9, 105–120 (2004). 15009096 10.1111/j.1365-2443.2004.00705.x

[b22] Perpelescu M., Nozaki N., Obuse C., Yang H. & Yoda K. Active establishment of centromeric CENP-A chromatin by RSF complex. J. Cell Biol. 185, 397–407 (2009). 19398759 10.1083/jcb.200903088 PMC2700388

[b23] Helfricht A. . Remodeling and spacing factor 1 (RSF1) deposits centromere proteins at DNA double-strand breaks to promote non-homologous end-joining. Cell Cycle 12, 3070–3082 (2013). 23974106 10.4161/cc.26033 PMC3875681

[b24] Pessina F. & Lowndes N. F. The RSF1 histone-remodelling factor facilitates DNA double-strand break repair by recruiting centromeric and Fanconi Anaemia proteins. PLoS Biol. 12, e1001856 (2014). 24800743 10.1371/journal.pbio.1001856 PMC4011676

[b25] Santamaria A. . The Plk1-dependent phosphoproteome of the early mitotic spindle. Mol. Cell. Proteomics 10, M110 004457 (2011). 20860994 10.1074/mcp.M110.004457 PMC3013462

[b26] Elia A. E. . The molecular basis for phosphodependent substrate targeting and regulation of Plks by the Polo-box domain. Cell 115, 83–95 (2003). 14532005 10.1016/s0092-8674(03)00725-6

[b27] Elia A. E., Cantley L. C. & Yaffe M. B. Proteomic screen finds pSer/pThr-binding domain localizing Plk1 to mitotic substrates. Science 299, 1228–1231 (2003). 12595692 10.1126/science.1079079

[b28] Cheng K. Y., Lowe E. D., Sinclair J., Nigg E. A. & Johnson L. N. The crystal structure of the human polo-like kinase-1 polo box domain and its phospho-peptide complex. EMBO J. 22, 5757–5768 (2003). 14592974 10.1093/emboj/cdg558 PMC275415

[b29] Matsumura S., Toyoshima F. & Nishida E. Polo-like kinase 1 facilitates chromosome alignment during prometaphase through BubR1. J. Biol. Chem. 282, 15217–15227 (2007). 17376779 10.1074/jbc.M611053200

[b30] Macurek L. . Polo-like kinase-1 is activated by aurora A to promote checkpoint recovery. Nature 455, 119–123 (2008). 18615013 10.1038/nature07185

[b31] Lowery D. M., Mohammad D. H., Elia A. E. & Yaffe M. B. The Polo-box domain: a molecular integrator of mitotic kinase cascades and Polo-like kinase function. Cell Cycle 3, 128–131 (2004). 14712072

[b32] Reinhardt H. C. & Yaffe M. B. Phospho-Ser/Thr-binding domains: navigating the cell cycle and DNA damage response. Nat. Rev. Mol. Cell Biol. 14, 563–580 (2013). 23969844 10.1038/nrm3640

[b33] Neef R. . Choice of Plk1 docking partners during mitosis and cytokinesis is controlled by the activation state of Cdk1. Nat. Cell Biol. 9, 436–444 (2007). 17351640 10.1038/ncb1557

[b34] Van Horn R. D. . Cdk1 activity is required for mitotic activation of aurora A during G2/M transition of human cells. J. Biol. Chem. 285, 21849–21857 (2010). 20444701 10.1074/jbc.M110.141010 PMC2898447

[b35] Salimian K. J. . Feedback control in sensing chromosome biorientation by the Aurora B kinase. Curr. Biol. 21, 1158–1165 (2011). 21723127 10.1016/j.cub.2011.06.015 PMC3156581

[b36] Kang Y. H. . Self-regulated Plk1 recruitment to kinetochores by the Plk1-PBIP1 interaction is critical for proper chromosome segregation. Mol. Cell 24, 409–422 (2006). 17081991 10.1016/j.molcel.2006.10.016

[b37] Park E. J., Erikson R. L. & Lee K. S. A self-propelled biological process: Plk1-dependent, product-activated feedforward mechanism. Cell Cycle 10, 3411–3412 (2011). 22067708 10.4161/cc.10.20.17522 PMC3266172

[b38] Dai W. & Wang X. Grabbing Plk1 by the PBD. Mol. Cell 24, 489–490 (2006). 17188028 10.1016/j.molcel.2006.11.004

[b39] Goto H. . Complex formation of Plk1 and INCENP required for metaphase-anaphase transition. Nat. Cell Biol. 8, 180–187 (2006). 16378098 10.1038/ncb1350

[b40] Cooke C. A., Heck M. M. & Earnshaw W. C. The inner centromere protein (INCENP) antigens: movement from inner centromere to midbody during mitosis. J. Cell Biol. 105, 2053–2067 (1987). 3316246 10.1083/jcb.105.5.2053 PMC2114862

[b41] Ainsztein A. M., Kandels-Lewis S. E., Mackay A. M. & Earnshaw W. C. INCENP centromere and spindle targeting: identification of essential conserved motifs and involvement of heterochromatin protein HP1. J. Cell Biol. 143, 1763–1774 (1998). 9864353 10.1083/jcb.143.7.1763 PMC2175214

[b42] Jeyaprakash A. A. . Structure of a Survivin-Borealin-INCENP core complex reveals how chromosomal passengers travel together. Cell 131, 271–285 (2007). 17956729 10.1016/j.cell.2007.07.045

[b43] Xu Z. . INCENP-aurora B interactions modulate kinase activity and chromosome passenger complex localization. J. Cell Biol. 187, 637–653 (2009). 19951914 10.1083/jcb.200906053 PMC2806593

[b44] McKinley K. L. & Cheeseman I. M. Polo-like kinase 1 licenses CENP-A deposition at centromeres. Cell 158, 397–411 (2014). 25036634 10.1016/j.cell.2014.06.016 PMC4192726

[b45] Barnhart-Dailey M. C. & Foltz D. R. Centromere Licensing: Mis18 Is Required to Polo-ver. Curr. Biol. 24, R808–R810 (2014). 25202874 10.1016/j.cub.2014.07.026 PMC5881566

[b46] Kim Y. . A library of TAL effector nucleases spanning the human genome. Nat. Biotechnol. 31, 251–258 (2013). 23417094 10.1038/nbt.2517

[b47] Kim Y. H., Ramakrishna S., Kim H. & Kim J. S. Enrichment of cells with TALEN-induced mutations using surrogate reporters. Methods 69, 108–117 (2014). 24780521 10.1016/j.ymeth.2014.04.012

[b48] Chae S. . HBxAPalpha/Rsf-1-mediated HBx-hBubR1 interactions regulate the mitotic spindle checkpoint and chromosome instability. Carcinogenesis 34, 1680–1688 (2013). 23536579 10.1093/carcin/bgt105

[b49] Fjeldbo C. S. . Functional studies on transfected cell microarray analysed by linear regression modelling. Nucleic Acids Res. 36, e97 (2008). 18628295 10.1093/nar/gkn428 PMC2528170

[b50] Kim J. S. . The auto-ubiquitylation of E3 ubiquitin-protein ligase Chfr at G2 phase is required for accumulation of polo-like kinase 1 and mitotic entry in mammalian cells. J. Biol. Chem. 286, 30615–30623 (2011). 21768102 10.1074/jbc.M111.231803 PMC3162422

