# Supplementary material for: The chromatin remodeller RSF1 is essential for PLK1 deposition and function at mitotic kinetochores
Source: Nat Commun. 2015 Aug 10;6:7904. doi: 10.1038/ncomms8904 (PMC4918322; doi:10.1038/ncomms8904)
Supplement: Supplementary Information — Supplementary Figures 1-8 [file ncomms8904-s1.pdf]

Supplementary Figure 1

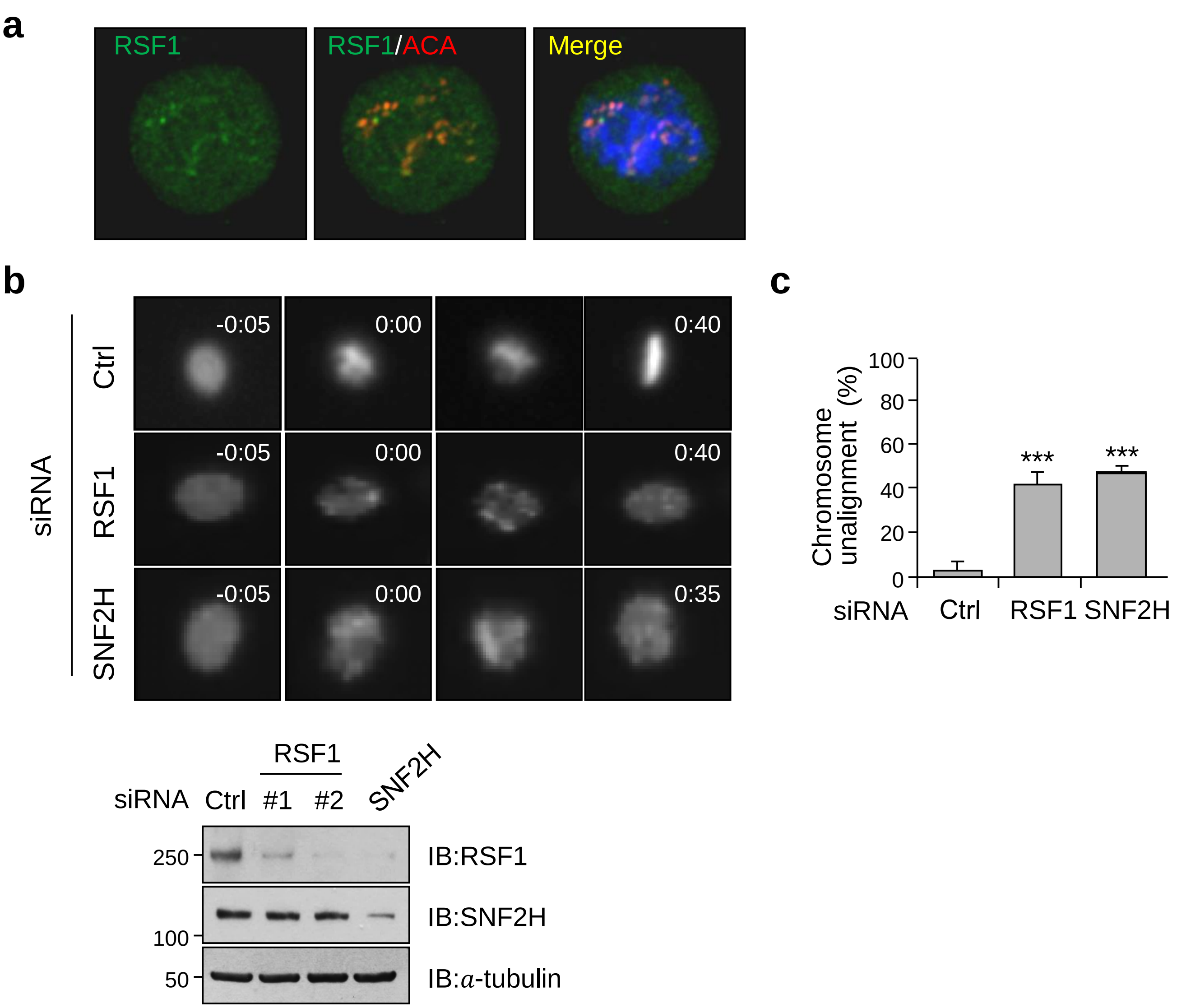

Supplementary Figure 1. RSF1 locates at mitotic kinetochores and it's depletion leads to defects in chromosome alignment.

(a) HeLa cells were fixed and stained with anti-PLK1 (green), anti-ACA (red) and DAPI (blue). (b) HeLa cells were mock transfected or transfected with siRNA against RSF1 along with the H2B-GFP plasmid. The cells were synchronized at the G_1/S boundary by adding 2 mM of thymidine for 24 h and released from the thymidine block for 8 h. Images were acquired every 5 min and the first chromosome condensation was set to zero time-point. The Immunoblot shows efficient reduction of RSF1 and SNF2H protein levels. (c) The quantification of unaligned chromosomes (%) from the acquired images of (b). *** $p < 0.001$ by Student's *t-test.* Data are represented as means ± s.e.m. (n=3).

Supplementary Figure 2

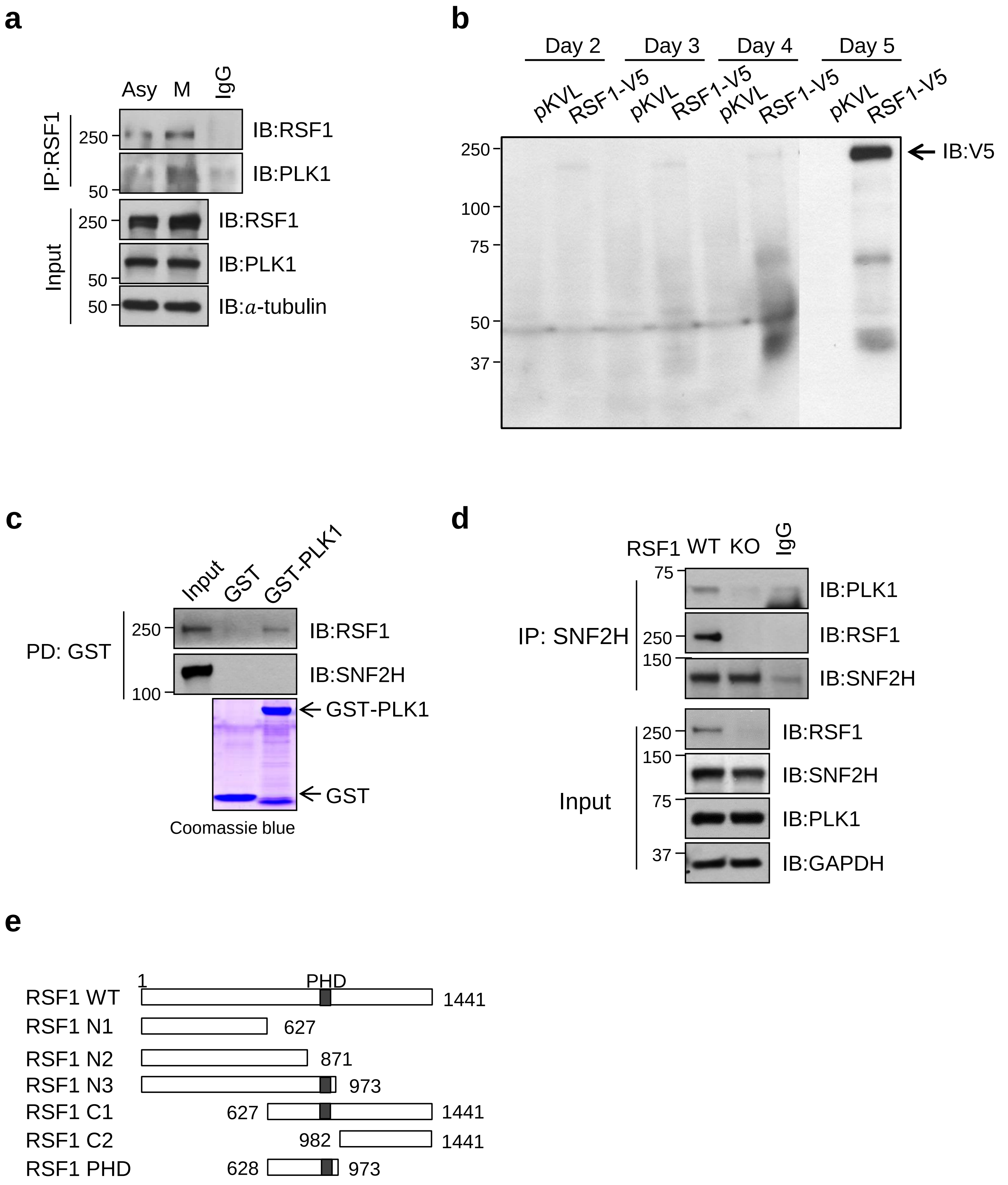

Supplementary Figure 2. RSF1 associates with PLK1.

(a) HeLa cell lysates were obtained from asynchronously growing cells (Asy) and mitotic cells (M) after treatment of nocodazole for 12 h. The lysates were immunoprecipitated with anti-RSF1 antibody (endogenous) or mouse IgG antibody and followed by immunoblotting with indicated antibodies. (b) The immunoblotting of purified RSF1-V5 protein. RSF1 gene was sublcloned into the pKVL plasmid that contains a ER leader sequence. Two µg of the plasmid encoding RSF1 was transfected to 100 mL of FreeStyle™ 293-F cells. After 2~5 days, the culture supernatant was harvested by a centrifugation and expression of RSF1-V5 protein was determined by immunoblotting. (c) Recombinant GST-PLK1 proteins were incubated with mitotic cell lyastes and subjected to immunoblotting. (d) HeLa cell lysates were obtained from RSF1 WT and RSF1 KO cells after treatment of paclitaxel for 12 h. The lysates were immunoprecipitated with anti-SNF2H antibody (endogenous) or mouse IgG antibody and followed by immunoblotting with indicated antibodies. (e) Schematic drawings of RSF1 deletion mutants used in the manuscript. See full blots Supplementary Fig. 8.

Supplementary Figure 3

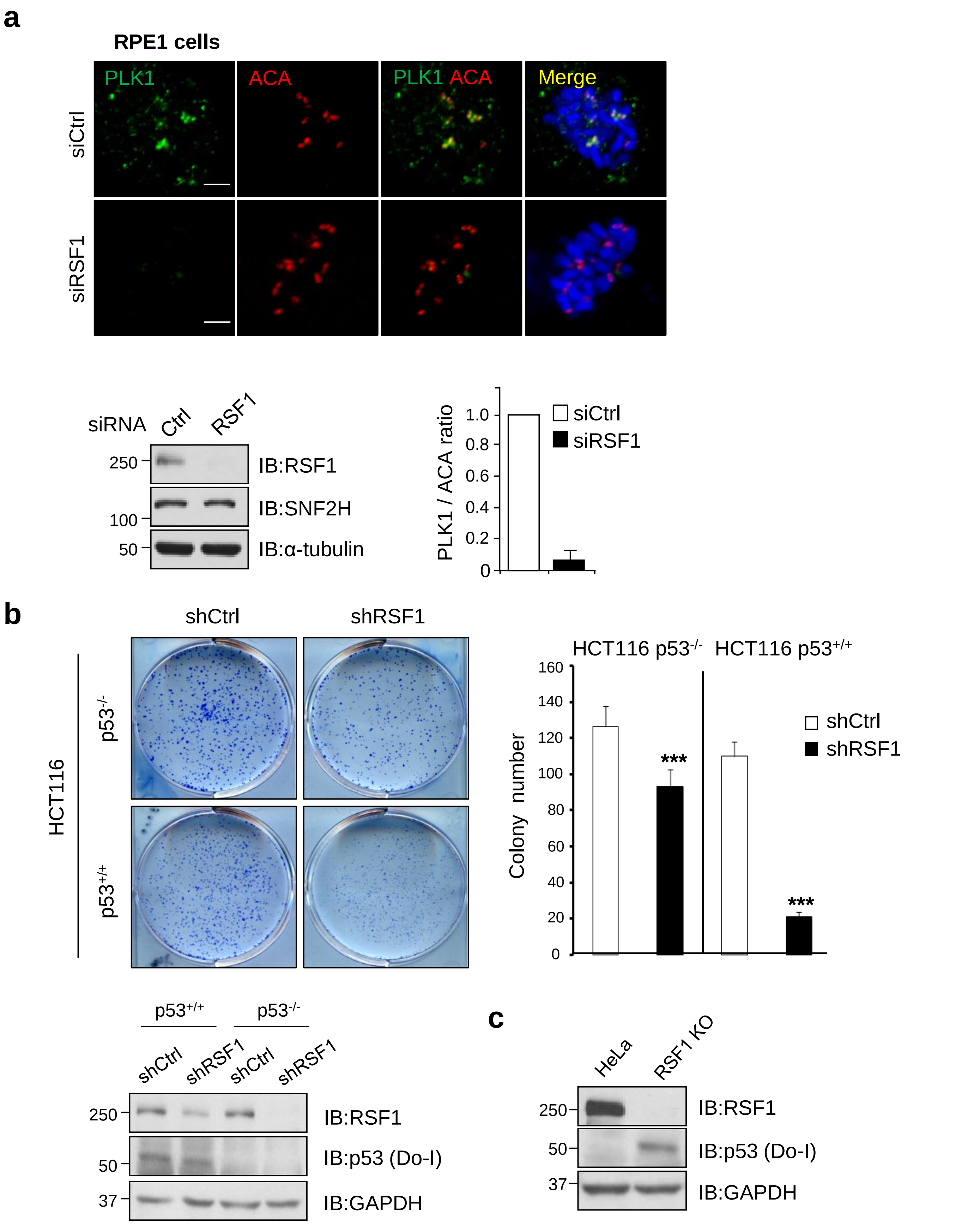

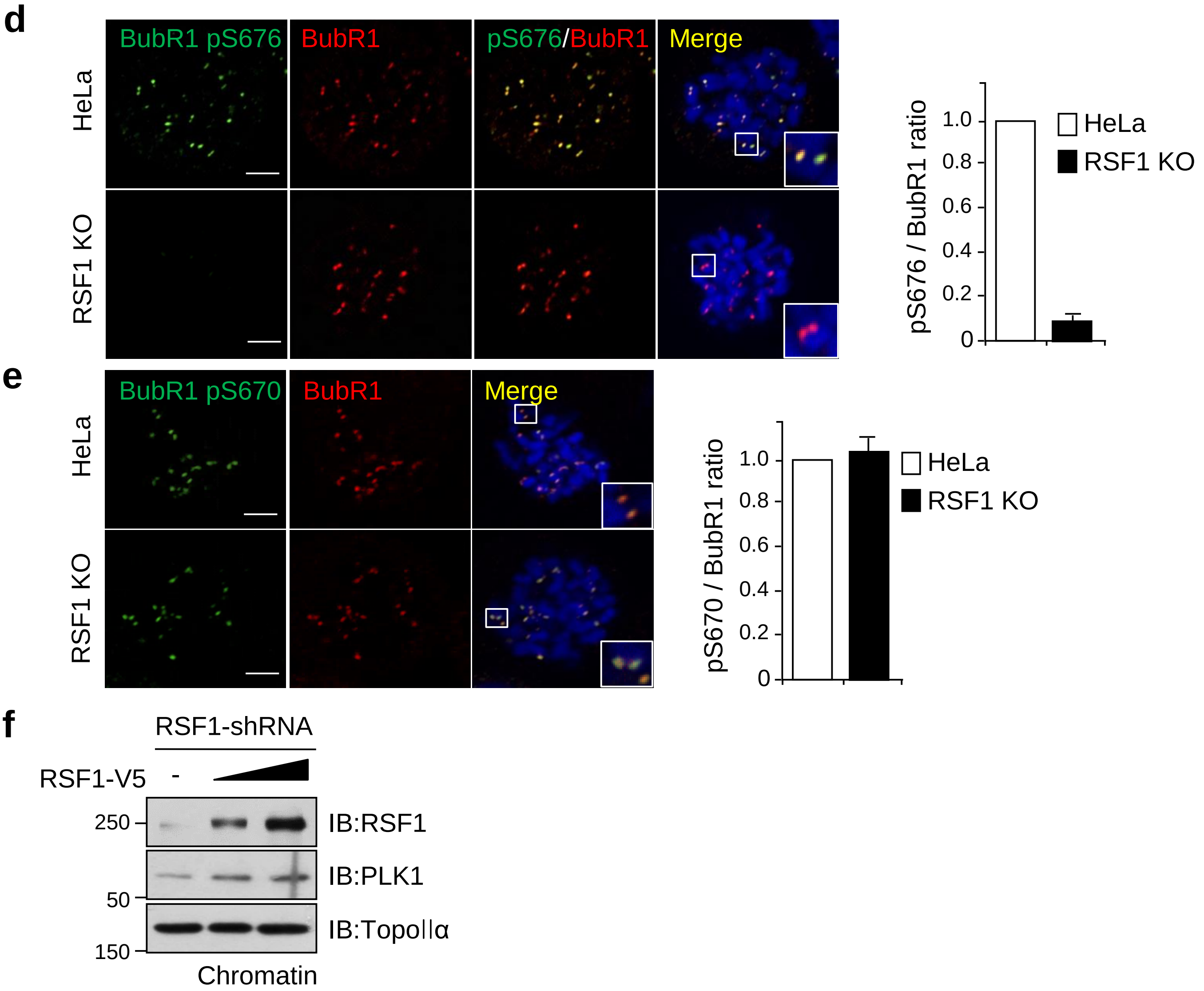

Supplementary Figure 3. RSF1 depletion hampers PLK1 recruitment to kinetochores.

(a) Human retinal pigment epithelial cells (RPE1) were either mock transfected or siRNA against RSF1 and treated with nocodazole for 4 h. The cells were subjected to immunostaining with anti-PLK1 (green) and anti-ACA (red) antibodies. Nucleus was stained with DAPI (blue). The Immunoblot shows reduction of RSF1 protein levels. Scale bar, 5 μm. (b) HCT116 cells stably transduced with a control or RSF1 shRNA were seeded in six-well plates ($3X10^3$), and were stained with crystal violet 7 days later. Wells are representative of three independent experiments for each cell line. The graph represents the quantification analysis of colony formation. *** $p < 0.001$ by Student's *t-test.* Data are represented as means ± s.e.m. (n=3). (c) Immunoblotting of p53 levels in RSF1 WT or KO HeLa cells (See full blots Supplementary Fig. 8.). (d,e) Depletion of RSF1 reduced the phosphorylation at Ser676 of BubR1, but not Ser670. HeLa cells and RSF1 KO HeLa cells were obtained after nocodazole treatment for 4 h and subjected to immunostaining. The phospho-specific antibodies against BubR1 (green) and total BubR1 (red) were applied for the comparison. Scale bar, 5 μm. Data are represented as means ± s.e.m. (n=3). (f) The HeLa cells stably expressing RSF1 shRNA targeting 3'UTR of RSF1 gene was established by infection of the lentiviral vector containing RSF1 shRNA, followed by clonal selection in the presence of puromycin (1 μg/ml). The RSF1-shRNA cells were transfected with RSF1-V5 constructs for 48 h and the chromatin fractions were obtained after centrifugation and wash with 0.5 M NaCl. The chromatin -bound PLK1 levels were determined by immunoblot.

Supplementary Figure 4

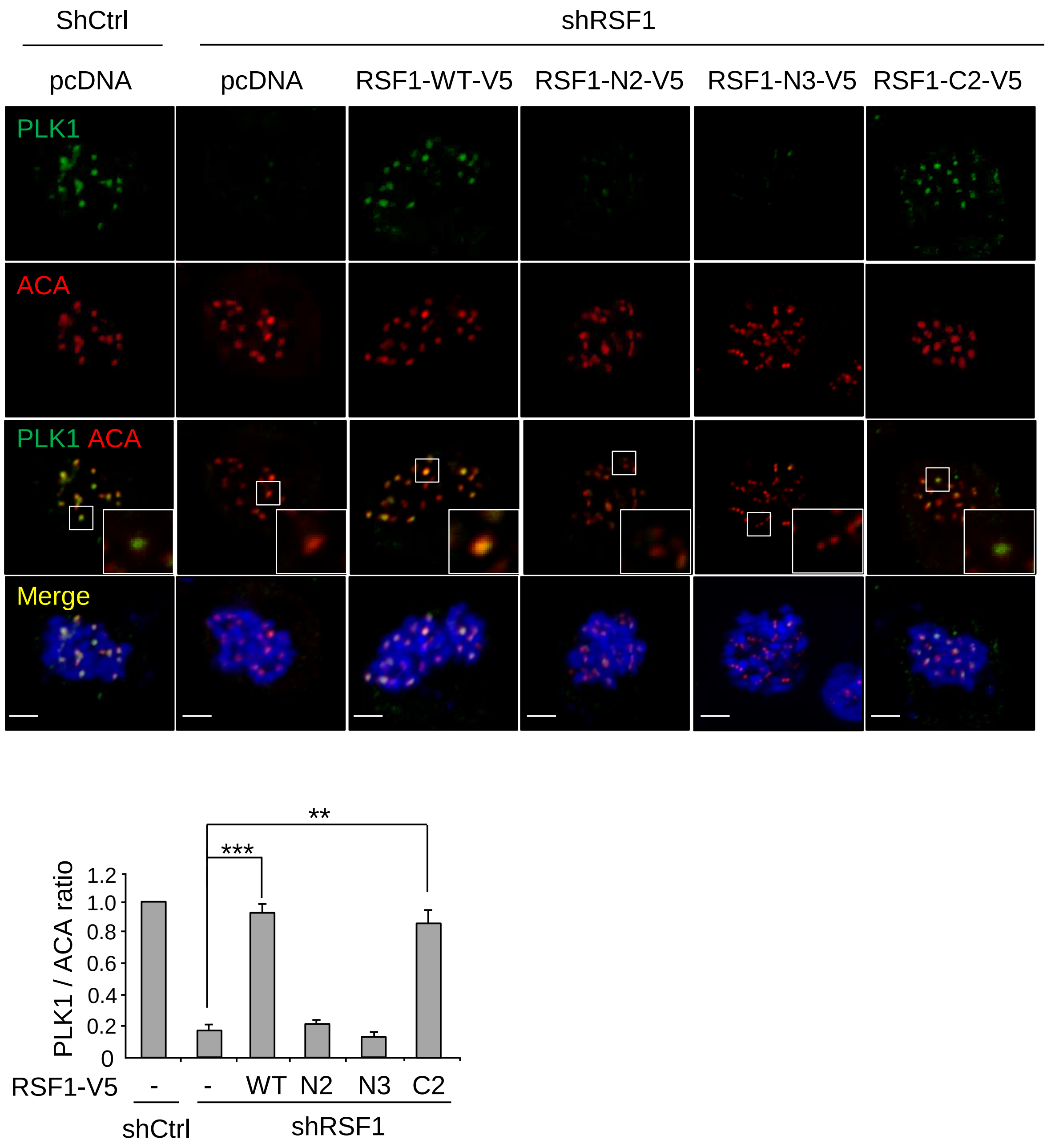

Supplementary Figure 4. RSF1 constructs retaining the PLK1 binding restored the PLK1 recruitment to kinetochores in RSF1 shRNA cells.

The RSF1 shRNA cells stably expressing RSF1 shRNA targeting 3'UTR of RSF1 gene were transfected with indicated V5 tagged RSF1 deletion mutant constructs. The cells were treated with nocodazole for 4 h, fixed and immunostained with indicated antibodies: PLK1 (anti-PLK1 antibody, green), RSF1(anti-RSF1 antibody, red), Nucleus (DAPI, blue). Scale bar, 5 μm. ** $p < 0.05$, *** $p < 0.001$ by Student's *t-test.* Data are represented as means ± s.e.m. (n=3).

Supplementary Figure 5

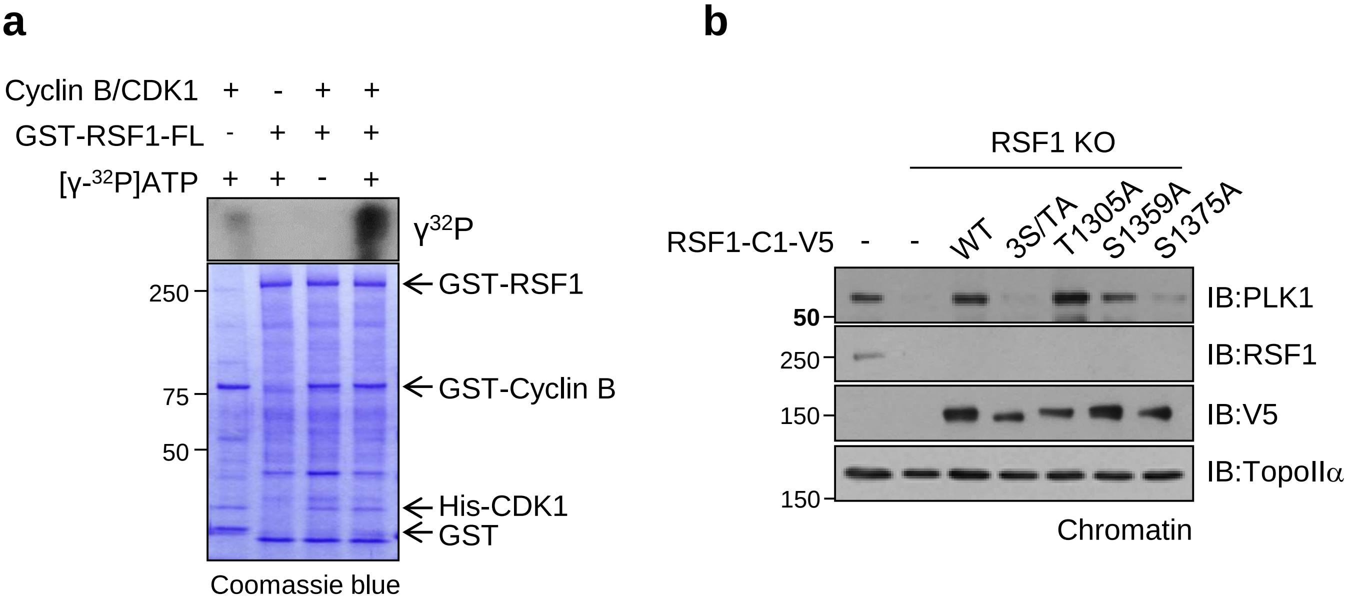

Supplementary Figure 5. RSF1 is phosphorylated by cyclin B/CDK1.

(a) RSF1 is phosphorylated by the cyclin B/CDK1 complex. The purified cyclin B and CDK1 proteins were incubated with the purified GST-RSF1-FL protein in the presence/absence of [γ-^{32}P]ATP. The mixture was subjected to electrophoresis and phosphorylated proteins by CDK1 were visualized by autoradiography. (b) RSF1-C1 mutant plasmids were transfected into RSF1 KO cells and treated with paclitaxel for 12 h. The chromatin-bound fractions were obtained after centrifugation and wash with 0.5 M NaCl, and subjected to immunoblot analysis with indicated antibodies. TopoIIα was used as a control of the chromatin fraction. See full blots Supplementary Fig. 8.

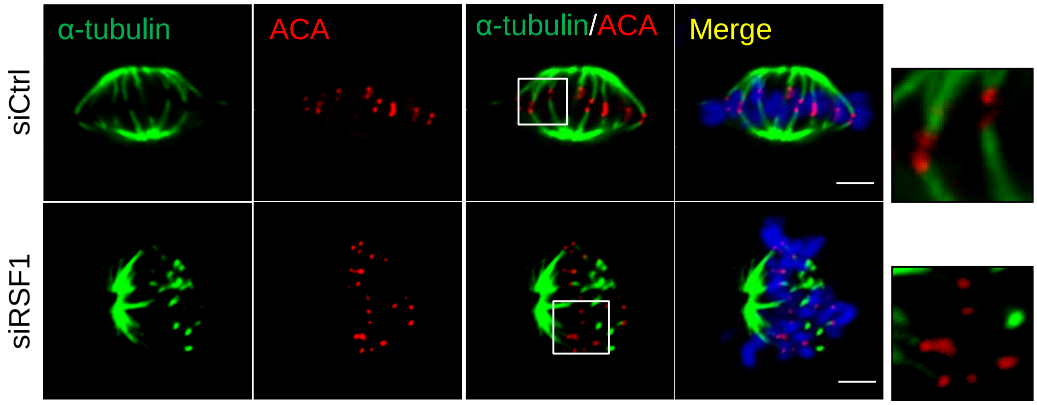

Supplementary Figure 6. Microtubule-kinetochore attachments are defective in RSF1 depleted cells.

HeLa cells were transfected with scrambled siRNA or RSF1 siRNA for 48 h. The cells were then incubated at 4 °C for the 10 min before fixation. Immunofluorescence images were visualized under confocal microscope using tubulin (green) and ACA (red) antibodies. Scale bar, 5 μm.

Supplementary Figure 7

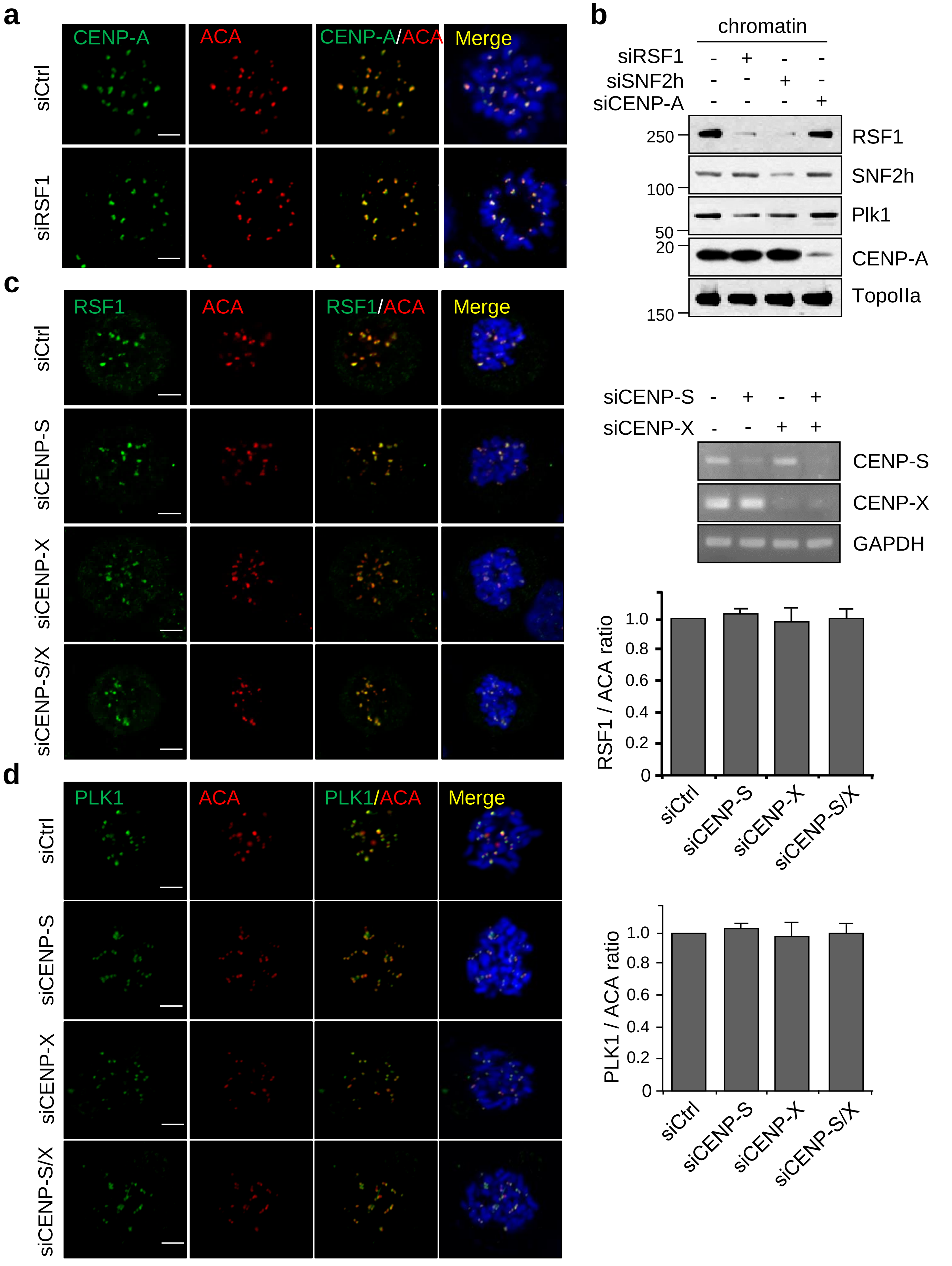

e

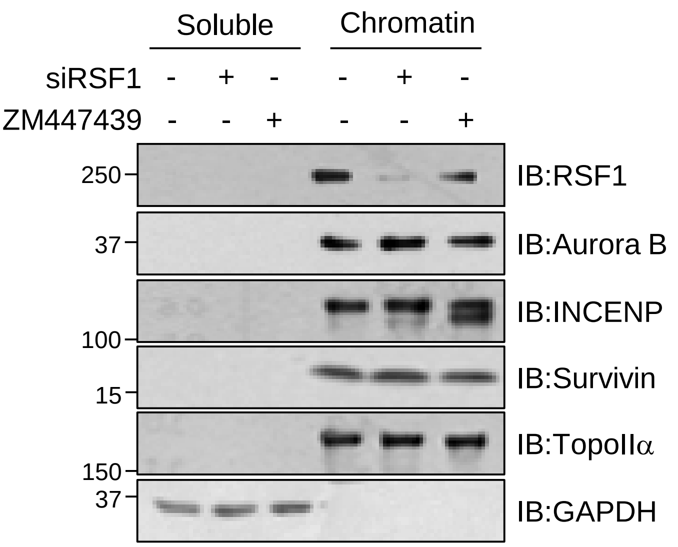

Soluble
Chromatin
siRSF1 - + - - + -
ZM447439 - - + - - +
250
37
100
15
150
37
IB:RSF1
IB:Aurora B
IB:INCENP
IB:Survivin
IB:TopoIIα
IB:GAPDH

f

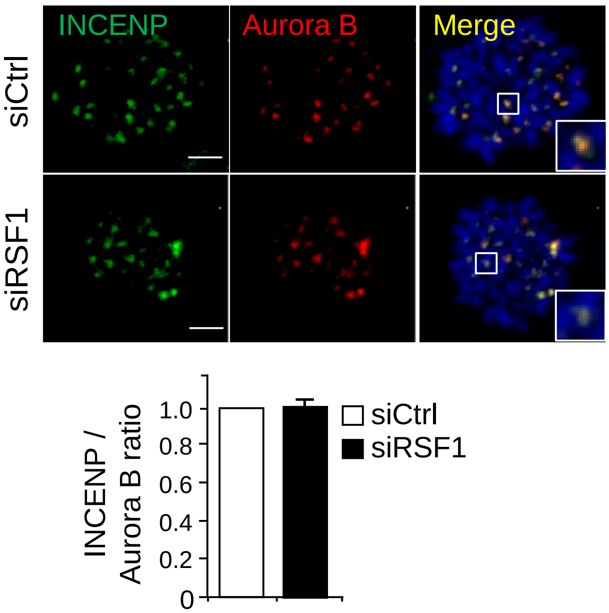

INCENP
Aurora B
Merge
siCtrl
siRSF1
INCENP / Aurora B ratio
1.0
0.8
0.6
0.4
0.2
0
siCtrl
siRSF1

g

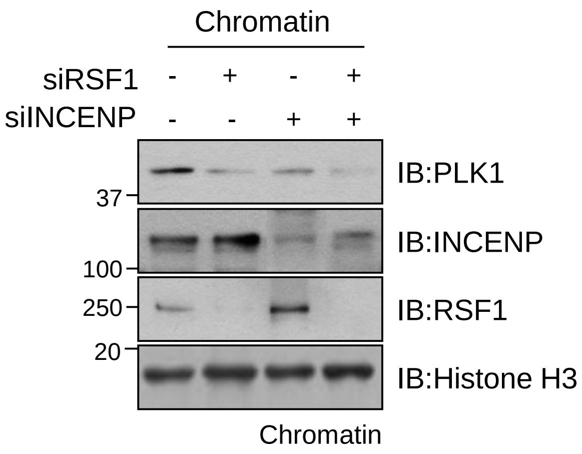

Chromatin
siRSF1 - + - +
siINCENP - - + +
37
100
250
20
IB:PLK1
IB:INCENP
IB:RSF1
IB:Histone H3
Chromatin

h

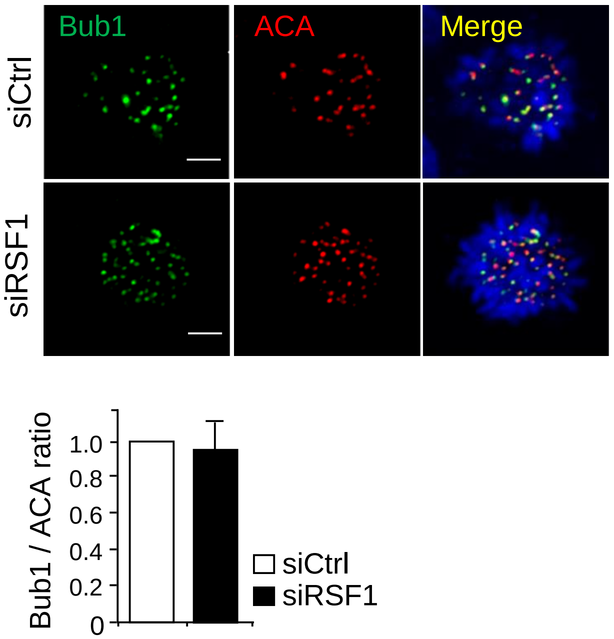

Bub1
ACA
Merge
siCtrl
siRSF1
Bub1 / ACA ratio
1.0
0.8
0.6
0.4
0.2
0
siCtrl
siRSF1

i

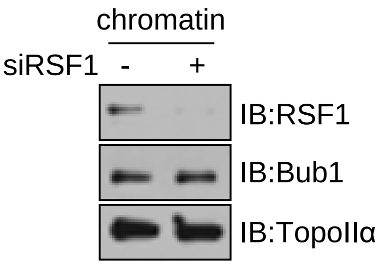

chromatin
siRSF1 - +
IB:RSF1
IB:Bub1
IB:TopoIIα

Supplementary Figure 7. Kinetochore localization of RSF1 and PLK1 is not related to centromere proteins.

(a) HeLa cells that were mock transfected or transfected with siRNA against RSF1 were synchronized at mitosis by nocodazole treatment. Immunofluorescence images of CENP-A (green), ACA (red) and nucleus (blue) were shown. Scale bar, 5 μm. (b) HeLa cells that were transfected with siRNA against RSF1 or CENP-A were synchronized at mitosis by paclitaxel treatment. The chromatin-bound fraction were analyzed by immunoblot analysis with indicated antibodies. (c,d) HeLa cells that were mock transfected or transfected with siRNA against CENP-S or CENP-X were synchronized at mitosis by nocodazole treatment. Immunofluorescence images of RSF1 or PLK1 (green), ACA (red) and nucleus (blue) were shown. The RT-PCR at the right panel shows reduction of CENP-S or CENP-X protein levels. Scale bar, 5 μm. (e,f) HeLa cells that were mock transfected or transfected with siRNA against RSF1 were synchronized at mitosis by Paclitaxel treatment. Before harvest, Aurora B inhibitor, ZM447429 was added to the cells for 20 min. Proteins eluted as soluble fraction and the chromatin-bound fraction were analyzed by immunoblot analysis with indicated antibodies (e) or by immunostaining (f) Immunofluorescence images of INCENP (green), Aurora B (red) and nucleus (blue) were shown. Scale bar, 5 μm. (g) Depletion of RSF1 or INCENP reduced the chromatin bound PLK1 levels. HeLa cells that were transfected with siRNA against RSF1 or INCENP were synchronized at mitosis by paclitaxel treatment. The chromatin-bound fraction were analyzed by immunoblot analysis with indicated antibodies. Scale bar, 5 μm. (h,i) HeLa cells that were mock transfected or transfected with siRNA against RSF1 were synchronized at mitosis by nocodazole treatment. Immunofluorescence images of Bub1 (green), ACA (red) and nucleus (blue) were shown (h). Proteins eluted as the chromatin-bound fraction were analyzed by immunoblot analysis with indicated antibodies (i). Data are represented as means $\pm$ s.e.m. (n=3).

Supplementary Figure 8

Figure 1 b

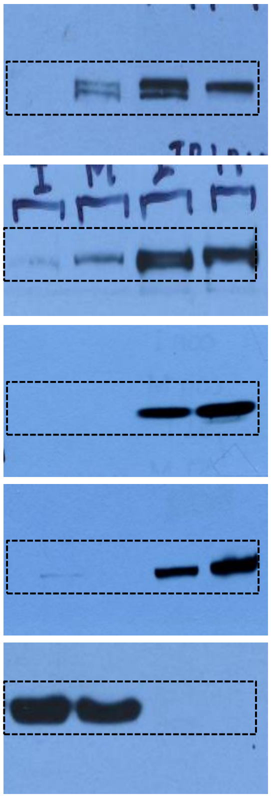

Figure 1 d

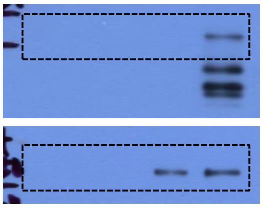

Figure 1 e

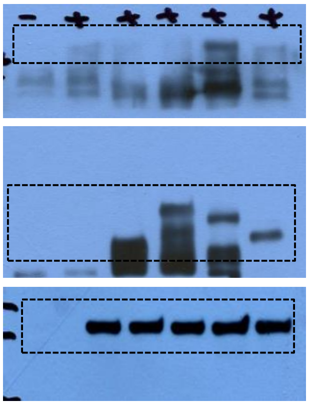

Figure 1 f

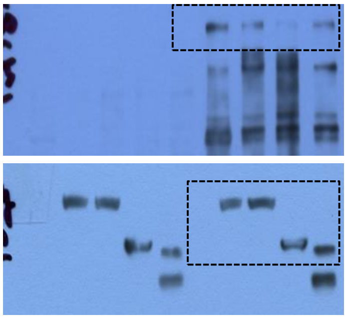

Figure 1 h

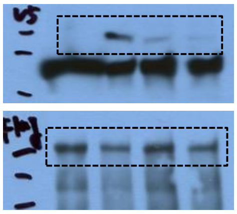

Figure 1 g

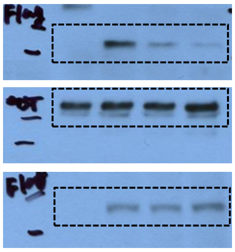

Figure 2 b

WCL Chromatin

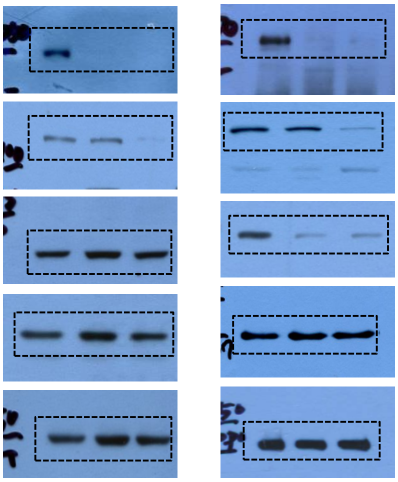

Figure 2 e

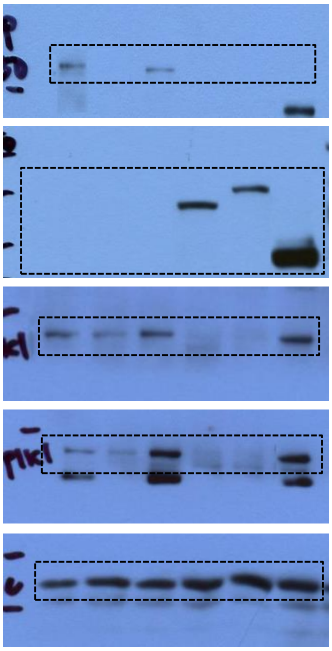

Figure 3 a

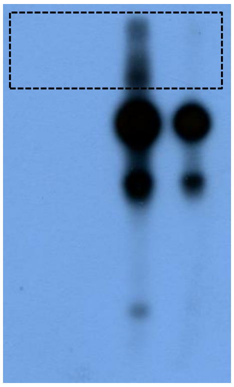

Figure 3 b

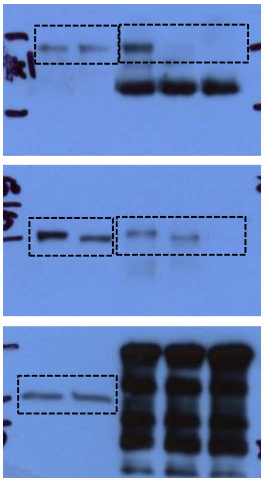

Figure 3 c

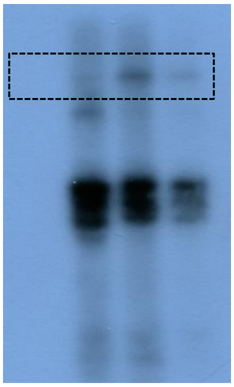

Figure 3 d

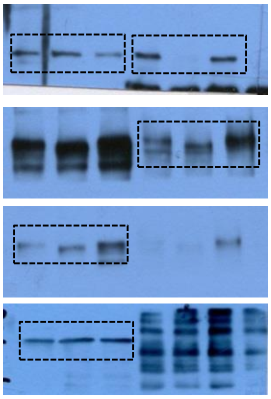

Figure 4 a

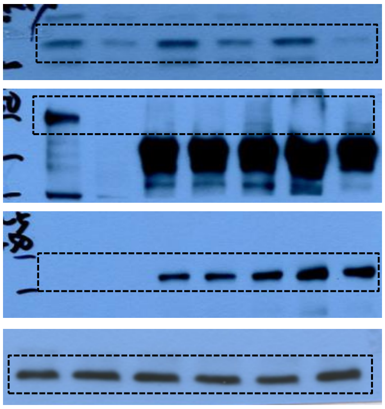

Figure 4 b

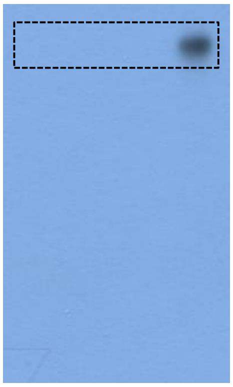

Figure 4 c

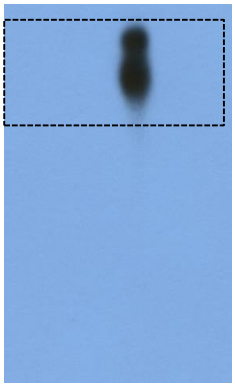

Figure 4 d

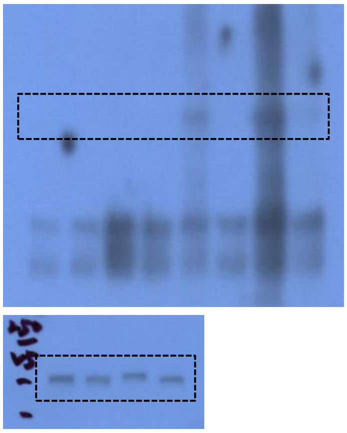

Figure 4 e

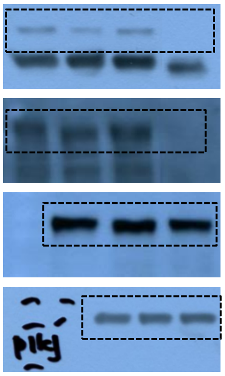

Supplementary Figure 2 d

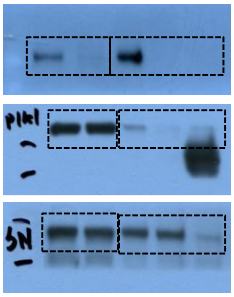

Supplementary Figure 2 e

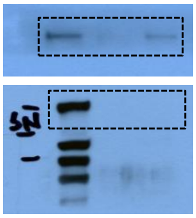

Supplementary Figure 3 c

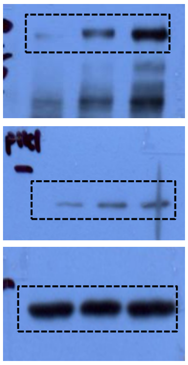

Supplementary Figure 5 a

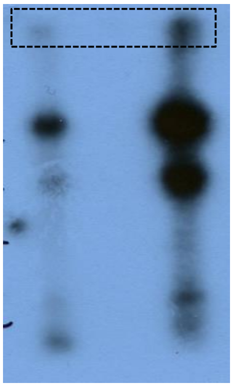

Supplementary Figure 5 b

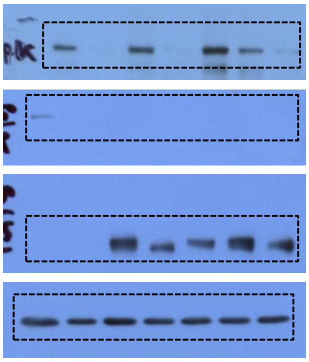

Supplementary Figure 8. Full blots for immunoblotting and *in vitro* kinase assay shown in figures and supplementary figures.
